# Impact of adverse childhood experiences on analgesia-related outcomes: a systematic review

**DOI:** 10.1016/j.bja.2024.09.015

**Published:** 2024-10-22

**Authors:** Dhaneesha N.S. Senaratne, Mia Koponen, Karen N. Barnett, Blair H. Smith, Tim G. Hales, Louise Marryat, Lesley A. Colvin

**Affiliations:** 1 Chronic Pain Research Group, Division of Population Health and Genomics, School of Medicine, University of Dundee, Dundee, UK; 2 Institute of Academic Anaesthesia, Division of Systems Medicine, School of Medicine, University of Dundee, Dundee, UK; 3 School of Health Sciences, University of Dundee, Dundee, UK

**Keywords:** addiction, adverse childhood experiences, childhood trauma, early life adversity, opioid use disorder

## Abstract

**Background:**

There is well-established evidence linking adverse childhood experiences (ACEs) and chronic pain in adulthood. It is less clear how ACE exposure might influence the response to chronic pain treatment. In this systematic review, we synthesise the literature assessing the impact of ACE exposure on outcomes relating to the use, benefits, and harms of analgesic medications (analgesia-related outcomes).

**Methods:**

We searched seven databases from inception to September 26, 2023, for studies investigating adverse events in childhood (<18 yr) and any analgesia-related outcome during adulthood (≥18 yr). Title/abstract screening, full-text review, data extraction, and risk of bias assessment were performed independently by two authors. Given the high degree of study heterogeneity, a narrative synthesis was performed.

**Results:**

From 7531 records, 66 studies met inclusion criteria, involving 137 395 participants. Analgesia-related outcomes were classed into six categories: use of analgesics (*n*=12), analgesic side-effects (*n*=4), substance misuse (*n*=45), lifetime drug overdose (*n*=2), endogenous pain signalling (*n*=4), and other outcomes (*n*=2). No studies assessed the effect of ACE exposure on the potential benefits of analgesics. ACE exposure was associated with greater use of analgesic medication, higher incidence of analgesic medication side-effects, greater risk and severity of substance misuse, greater risk of drug overdose, and greater risk of attempted suicide in opioid dependency.

**Conclusions:**

Adverse childhood experience exposure is associated with poor analgesia-related outcomes, so individual assessment adverse childhood experiences is important when considering the treatment of chronic pain. However, significant gaps in the literature remain, especially relating to the use and harms of non opioid analgesics.

**Systematic review protocol:**

CRD42023389870 (PROSPERO).

**Editor's key points**

• Adverse childhood experiences (ACEs) are linked to chronic pain in adulthood, but the consequences of this for clinical practice are not clear.
• In this systematic review, the authors found that ACEs were linked to poor outcomes after analgesia (predominantly opioids) and that evidence relating to the benefits of medication (e.g. effectiveness) was missing.
• Routine ACE assessment may be useful in settings such as the chronic pain clinic, but significant evidence gaps remain.

Adverse childhood experiences (ACEs) are potentially stressful events or environments that occur before the age of 18 yr. They can be considered in terms of abuse (e.g. physical or sexual), neglect (e.g. emotional or physical), household challenges (e.g. parental separation or household member mental illness), and external challenges (e.g. bullying or war). ACEs are common, with estimates suggesting that 47% of the UK adult population have experienced at least one type and 12% have experienced four or more.^1^

A large body of epidemiological data has linked ACE exposure to a range of poor physical and mental health outcomes in adulthood, with a dose–dependent relationship.^1^, ^2^, ^3^, ^4^, ^5^, ^6^, ^7^, ^8^, ^9^ Several mechanisms have been proposed to underpin these relationships, including epigenetic changes,^10^^,^^11^ alterations to stress signalling pathways,^12^, ^13^, ^14^ and differences in the structure and function of the brain.^15^, ^16^, ^17^, ^18^ Collectively, these mechanisms contribute to, or are outcomes of, ‘toxic stress’; this is the concept that cumulative stress experienced at important developmental stages can lead to biological and behavioural changes that may cause long-term harm.^19^

Chronic pain is defined as pain that persists or recurs for longer than 3 months, that is, beyond the time expected for normal tissue healing.^20^ It is also common: overall adult prevalence estimates range from 19% to 44% with higher prevalence in females and older people.^21^, ^22^, ^23^, ^24^ There is a strong body of evidence to support the association between ACE exposure and the prevalence of adult chronic pain; this link is consistent in a range of different populations and demonstrates a dose–dependent relationship.^25^, ^26^, ^27^, ^28^, ^29^, ^30^, ^31^, ^32^ For example, a recent meta-analysis reported that exposure to any ACE was associated with 53% higher odds of experiencing a chronic painful condition in adulthood and 29% higher odds of experiencing pain-related disability.^32^

The most appropriate pharmacological management of chronic pain is debated, especially as some of the commonly used drug classes have the potential to cause harm. Opioids are of particular concern given recent increases in medical and nonmedical prescription opioid use and the associated media spotlight on the opioid crisis.^33^, ^34^, ^35^ Systematic reviews report reasonable evidence for opioid effectiveness in acute pain and cancer pain^36^; however, their role in treating chronic non-cancer pain is less clear.^37^^,^^38^ As opioid use rises, so too do adverse opioid-related outcomes such as addiction, overdose, and death.^34^^,^^39^, ^40^, ^41^, ^42^, ^43^ There is a trend towards deprescribing opioids in people with chronic non-cancer pain, but this can be challenging to implement in practice.^44^^,^^45^ The trend for gabapentinoids is similar, although less well documented; there is reasonable evidence for their effectiveness in chronic neuropathic pain but less so for other forms of chronic pain.^46^^,^^47^ Prescription rates of gabapentinoids and gabapentinoid-associated deaths are also both increasing, as are associated harms.^34^^,^^43^^,^^48^, ^49^, ^50^

How a history of ACE exposure might influence the pharmacological management of chronic pain is less well established. Animal models of early life adversity, primarily modelling neglect in the form of maternal deprivation, have demonstrated an impact on nociception and responses to opioids, including the development of addiction-like behaviours.^51^, ^52^, ^53^ Synthesising the evidence in humans is an important step in understanding the impact of ACEs and how we might mitigate their effects. In this review, we focused on pharmacological management and aimed to assess whether exposure to ACEs is related to the use, benefits, and harms of analgesic medications (analgesia-related outcomes). Answers to this question could provide clearer information to healthcare practitioners and patients to inform chronic pain treatment discussions.

## Methods

### Search strategy and selection criteria

We conducted a systematic review that was registered in the International Prospective Register of Systematic Reviews (PROSPERO) on September 15, 2023 (CRD42023389870). We reported following the Preferred Reporting Items for Systematic Reviews and Meta-Analyses (PRISMA) guidelines.

We developed a search strategy based on previously published literature reviews and refined it following input from subject experts, an academic librarian, and our patient and public partners (Supplementary Table S1). The strategy included strings for ACEs, generic analgesia terms, specific analgesic groups, and 59 individual analgesics taken from relevant sections of the British National Formulary.^54^ The strategy did not include strings for any specific outcome to allow identification of all possible outcomes. We searched the following seven databases from inception to September 26, 2023: APA PsycNET, CINAHL Plus, Cochrane CENTRAL, Embase, MEDLINE, Scopus, and Web of Science. The search results were imported into Covidence (Veritas Health Innovation, Melbourne, VIC, Australia), which automatically identified and removed duplicate entries. Two reviewers (DS and MK) independently performed title/abstract screening (inter-rater reliability using Cohen's kappa = 0.45) and full-text review (Cohen's kappa = 0.90). Discrepancies were resolved by consensus discussion.

Reports were eligible for review if they included adults (≥18 yr), adverse events that had occurred during childhood (<18 yr), and any analgesia-related outcome. Reports that only assessed adverse events in adulthood or analgesia-related outcomes in children were excluded. The following study designs were eligible: randomised controlled trials, cohort studies, case–control studies, cross-sectional studies, and review articles with meta-analysis. Editorials, case reports, and conference abstracts were excluded. Systematic reviews without a meta-analysis and narrative synthesis review articles were also excluded; however, their reference lists were screened for relevant citations.

### Data analysis

Two reviewers (DS and either MK or KB) independently performed data extraction into Microsoft Excel (Microsoft Corporation, Redmond, WA, USA) using a pre-agreed template. Discrepancies were resolved by consensus discussion. Data extracted from each report included study details (author, year, study design, sample cohort, sample size, and sample country of origin), patient characteristics (age and sex), ACE information (definition, childhood cut-off age, number of ACEs, list of ACEs, and ACE prevalence), analgesia-related outcome information (outcome measured and outcome definition), and analysis parameters (effect size and confidence intervals).

Two reviewers (DS and either MK or KB) independently performed risk of bias assessments of each included study using either the Risk Of Bias In Non-randomized Studies of Exposures (ROBINS-E) tool for observational studies or the Risk of Bias 2 (RoB 2) tool for randomised trials.^55^^,^^56^ The ROBINS-E tool assesses the risk of bias across seven domains: confounding; measurement of the exposure, participant selection, postexposure interventions, missing data, measurement of the outcome, and selection of the reported result. The RoB 2 tool assesses the risk of bias across five domains: randomisation, deviation from the intended intervention, missing outcome data, measurement of the outcome, and selection of the reported result. Discrepancies were resolved by consensus discussion.

We made no assumptions about the types of analgesia-related outcomes that could have been identified; however, we ultimately classed the included papers into the following six outcome categories: use of analgesics, analgesic side-effects, substance misuse, lifetime drug overdose, endogenous pain signalling, and other outcomes. When considering the harmful use of substances, we opted to use the term *substance misuse* rather than *substance use disorder* (or equivalent) as the latter implies that diagnostic criteria have been met, whereas the former encompasses a broader range of harmful scenarios.^57^

All statistical analyses were performed in R version 4.2.2 using the RStudio integrated development environment (RStudio Team, Boston, MA, USA). To avoid repetition of individual participant data, where multiple studies analysed the same patient cohort, we selected the study with the largest sample size. Meta-analysis of prevalence was performed with the meta package, using logit transformations within a generalised linear mixed model and reporting the random-effects model.^58^^,^^59^

### Patient and public involvement

This review had input from members of the Chronic Pain Advisory Group (CPAG), who are part of the Consortium Against Pain Inequality (CAPE).^60^ CPAG consists of eight individuals with lived experiences of ACEs and chronic pain. The group has experience in systematic review co-production and provided feedback on the choice of topic and framing of the research question.

## Results

**Fig 1:**
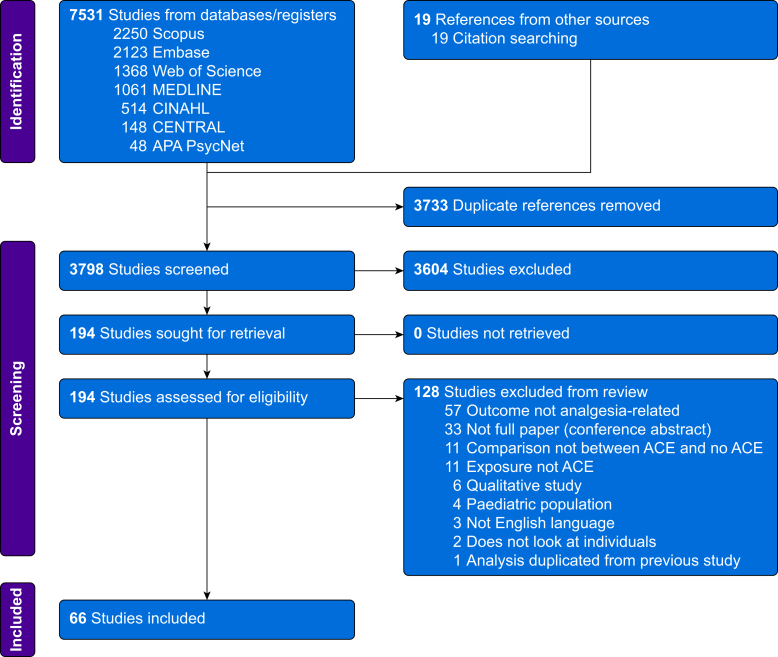
Flow chart of selection of studies into the systematic review. ACE, adverse childhood experience.

The search identified 7531 records, of which 66 met the inclusion criteria (Fig. 1). Sixty-five studies were observational, and one was a randomised controlled trial. The total participant count (discounting duplicated cohorts) was 137 395. The majority of studies were from North American (*n*=47, 71.2%), European (*n*=11, 16.7%), or Australian (*n*=6, 9.1%) populations; African and Middle Eastern populations were represented by one study each (1.5% each). The summary characteristics can be found in Supplementary Table S2.

### Risk of bias assessment

Risk of bias assessment was performed on 65 observational studies using the ROBINS-E tool (Supplementary Table S3).^55^ Overall most studies were at ‘high risk’ (*n*=21, 31.8%) or ‘very high risk’ (*n*=44, 66.7%) of bias.

There were some consistent risks observed across the studies, especially in domain 1 (risk of bias attributed to confounding) and domain 3 (risk of bias attributed to participant selection). In domain 1, most studies were ‘high risk’ or ‘very high risk’ (*n*=59, 89.4%) as either they performed unadjusted analysis, introducing a risk of confounding bias, or they controlled for variables that could have been affected by ACE exposure (e.g. coexisting mental health disorders), increasing the risk of overadjustment bias. In domain 3, many studies were ‘high risk’ or ‘very high risk’ (*n*=43, 65.2%) as participant selection was based on characteristics that could have been influenced by ACE exposure (e.g. recruitment of participants attending a healthcare service), introducing a risk of selection bias. The remaining studies were deemed as having ‘some concerns’ (*n*=23, 34.8%) as participant selection occurred at a time after ACE exposure, introducing a risk of survivorship bias.

Differences in risk of bias were seen in domain 2 (risk of bias attributed to exposure measurement) and domain 5 (risk of bias attributed to missing data). In domain 2, some studies were at ‘high risk’ as they used a narrow or atypical measure of ACEs (*n*=15, 22.7%); others were graded as having ‘some concerns’ as they used a broader but still incomplete measure of ACEs (*n*=30, 45.5%); and the remainder were at ‘low risk’ as they used an established or comprehensive list of ACEs (*n*=21, 31.8%). In domain 5, many studies were at ‘high risk’ or ‘very high risk’ as they failed to acknowledge or appropriately address missing data (*n*=43, 65.2%); a few were graded as having ‘some concerns’ (*n*=3, 4.5%) as they had a significant amount of missing data (>10% of exposure, outcome, or confounders) but mitigated this with appropriate strategies; and the remainder were at ‘low risk’ as they reported low levels (<10%) of missing data (*n*=20, 30.3%).

Risk of bias assessment was performed on one interventional study using the RoB 2 tool (Supplementary Table S4).^56^ This study was graded as having ‘some concerns’ overall because of concerns in domain 1 (risk of bias attributed to randomisation), and domain 5 (risk of bias attributed to selection of the reported result).

### Exposure: adverse childhood experiences

There were differences in the way that the concept of ACEs was defined and measured across the 66 studies (Supplementary Table S5). Twenty-two different terms were used (although most were variations on similar themes), with the most common being ‘adverse childhood experiences’ (n=22, 33.3%), ‘childhood trauma’ (*n*=11, 16.7%), and ‘childhood maltreatment’ (*n*=7, 10.6%). Nearly two-thirds of studies (*n*=42, 63.6%) did not provide a formal definition of their term of choice. The upper age limit for childhood ranged from <12 to <19 yr, with the most common being <18 (*n*=26, 39.4%), although over a third of studies (*n*=27, 40.9%) did not report the age cut-off used.

**Fig 2:**
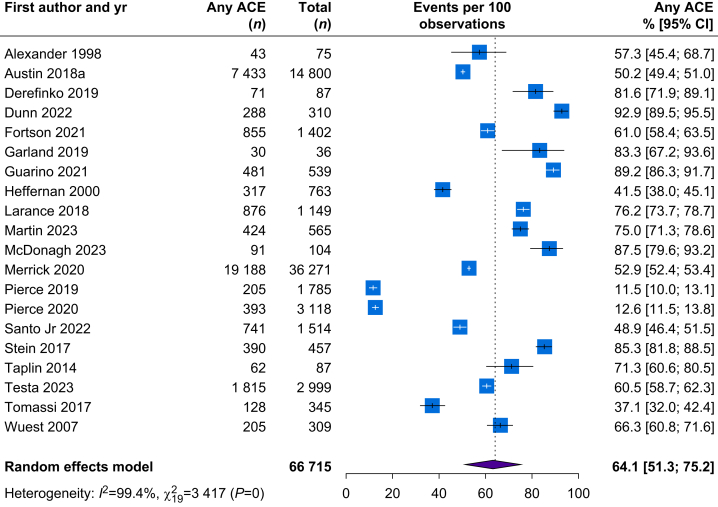
Meta-analysis of prevalence of exposure to any ACE. ACE, adverse childhood experience; CI, confidence interval.

In total, 45 different ACEs were assessed, with a median per study of 5 (range 1–27). The most frequently assessed ACEs were sexual abuse (*n*=55, 83.3%), physical abuse (*n*=52, 78.8%), and emotional abuse (*n*=44, 66.7%) (Supplementary Table S6). Twenty studies (30.3%) provided sufficient data to allow for a meta-analysis of the prevalence of exposure to any ACE; the pooled prevalence was 64.1% (95% confidence interval [CI] 51.3–75.2%). However, the inter-study heterogeneity was high (*I*^2^=99.4%, Cochran Q=3417, *P*<0.001) (Fig. 2).

Twenty-three studies (34.8%) compared their outcome of interest with individual ACE types (e.g. sexual or physical abuse). Twenty-eight studies (42.4%) used ACE counts (the sum of the different types of ACEs measured), 10 studies (15.2%) used ACE scores (incorporating ACE frequency or severity into the measurement), and five studies (7.6%) used other methods of ACE grouping (e.g. latent class analysis, ‘low’ *vs* ‘high’ trauma).

### Outcome: use of analgesics

Twelve studies (involving 27 281 participants) investigated the use of analgesic medication (Table 1).^61^, ^62^, ^63^, ^64^, ^65^, ^66^, ^67^, ^68^, ^69^, ^70^, ^71^, ^72^ Compared with adults with no/low ACE exposure, high ACE exposure was associated with a higher number of analgesic medications in 2/2 studies, use of over-the-counter analgesic medications in 1/1 study, use of any prescription analgesic medications in 1/2 studies, use of prescription opioids in 3/7 studies, and use of NSAIDs in 1/1 study, but not with use of benzodiazepines (1/1 study). Although four studies failed to show an association between ACEs and use of prescription opioids, these studies tended to have a smaller sample size (*n*=80, 113, 235, and 865) compared with the three studies that did show an association (n=230, 2999, and 14 800) and so may have been underpowered. The largest study, by Austin and colleagues,^62^ found that a history of any childhood abuse was associated with a 51% higher odds of recent prescription opioid use. We found no studies that examined whether ACE exposure influenced the potential benefits arising from use of analgesics (e.g. improvements in pain or functional status).

**Table 1:** Impact of adverse childhood experiences on the use of analgesics. ACE, adverse childhood experience; CI, confidence interval; NR, not reported; OR, odds ratio.

Author and yr	Participant population	Sample size	Exposure	Outcome	Outcome tool	Relationship
Alexander 1998	Women with fibromyalgia attending an outpatient rheumatology clinic	75	Sexual abuse and physical abuse	Medication usage	Number of pain medications	A history of abuse was associated with a higher number of pain medications (*t*-test, *P*=0.025).
Austin 2018a	National Longitudinal Study of Adolescent to Adult Health (Add Health), waves 1 and 4: a nationally representative cohort of adolescents aged 12–19 yr recruited from schools and followed up for more than 20 yr	14 800	Childhood abuse	Recent prescription opioid use	Structured interview asking about use of prescription opioids in the last 4 weeks	A history of any childhood abuse (logistic regression, OR 1.51, 95% CI 1.24–1.82), emotional abuse (logistic regression, OR 1.57, 95% CI 1.29–1.90), and physical abuse (logistic regression, OR 1.46, 95% CI 1.14–1.87) were associated with greater odds of recent prescription opioid use. A history of sexual abuse was not associated with greater odds of recent prescription opioid use (logistic regression, OR 1.14, 95% CI 0.72–1.80).
Baumann-Larsen 2023	Trøndelag Health Study (HUNT), Young-HUNT3 and Young-HUNT4: adolescents aged 13–19 yr living in the Nord-Trøndelag region (Young-HUNT3) followed up as adults (Young-HUNT4)	2947	Potentially traumatic events	Use of over-the-counter analgesics to treat musculoskeletal pain Use of over-the-counter analgesics to treat headache	Self-completed questionnaire asking about use of over-the-counter analgesics in the last month	Bullying (ordinal logistic regression, OR 1.84, 95% CI 1.28–2.66), physical violence (ordinal logistic regression, OR 1.53, 95% CI 1.07–2.19), sexual abuse (ordinal logistic regression, OR 1.70, 95% CI 1.12–2.57), witness to violence (ordinal logistic regression, OR 1.40, 95% CI 1.07–1.84), severe illness or death of someone close (ordinal logistic regression, OR 1.38, 95% CI 1.06–1.80), and severe accident, disaster, or other traumatic event (ordinal logistic regression, OR 1.40, 95% CI 1.07–1.84) were associated with use of over the counter analgesics to treat musculoskeletal pain. Bullying (ordinal logistic regression, OR 1.40, 95% CI 1.02–1.92), witness to violence (ordinal logistic regression, OR 1.28, 95% CI 1.03–1.59), and disease or death of someone close (ordinal logistic regression, OR 1.44, 95% CI 1.17–1.78) were associated with use of over-the-counter analgesics to treat headache. Physical violence (ordinal logistic regression, OR 1.23, 95% CI 0.90–1.68), sexual abuse (ordinal logistic regression, OR 1.23, 95% CI 0.85–1.77), and severe accident, disaster, or other traumatic event (ordinal logistic regression, OR 1.12, 95% CI 0.93–1.35) were not associated with the use of over-the-counter analgesics to treat headache.
Griego 2022	Women with chronic pelvic pain attending outpatient OB/GYN or pain clinics	113	ACEs	Used opioids in the last 3 months	NR	ACE count was not associated with use of opioids in the last 3 months (logistic regression, OR 1.05, 95% CI 0.92–1.19).
Lee 2023	Midlife in the United States study (MIDUS), wave 2: a nationally representative sample of adults	865	Childhood abuse	Opioid prescription	Medication review by study staff	Emotional abuse (logistic regression, OR 0.69, 95% CI 0.34–1.44), physical abuse (logistic regression, OR 0.45, 95% CI 0.19–1.02), and sexual abuse (logistic regression, OR 1.12, 95% CI 0.60–2.11) were not associated with opioid prescription.
Pierce 2019	Adults with current opioid use attending an outpatient pain clinic	1785	History of abuse	Current benzodiazepine use	Self-completed questionnaire asking about current benzodiazepine use	Childhood physical or sexual abuse was not associated with current benzodiazepine use (logistic regression, OR 1.08, 95% CI 0.63–1.84).
Sansone 2010	Adults attending an outpatient internal medicine clinic.	80	Childhood trauma	Total number of pain medications Narcotic pain medication use NSAID medication use Other pain medication use (gabapentin, duloxetine, and amitriptyline)	Review of the preceding 4 weeks of the medical record	A higher number of childhood trauma types was associated with a higher number of pain prescriptions (Pearson's correlation, *r*=0.34, *P*<0.0.1), higher NSAID medication use (Pearson's correlation, *r*=0.24, *P*<0.05), and higher other pain medication use (Pearson's correlation, *r*=0.28, *P*<0.05). The number of childhood trauma types was not associated with use of narcotic medications (Pearson's correlation, *r*=0.18, *P*>0.05).
Testa 2023	Pregnancy Risk Assessment Monitoring System (PRAMS), North Dakota and South Dakota populations: routine surveillance system for mothers who have recently given birth	2999	ACEs	Any prescription opioid use	Self-completed paper questionnaire or structured interview asking about prescription pain reliever use during the most recent pregnancy	≥3 ACEs was associated with higher odds of using prescription opioids during pregnancy (logistic regression; 0 ACEs: referent; 1 ACE: OR 1.88, 95% CI 0.98–3.64; 2 ACEs: OR 1.99, 95% CI 0.90–4.37; ≥3 ACEs: OR 2.44, 95% CI 1.32–4.50).
Williams 2020	Adults with a history of at least one type of interpersonal trauma (intimate partner violence, sexual assault, and/or ACEs) recruited through public advertising	230	ACEs	Prescription opioid use	Self-completed online questionnaire asking if they had received a prescription for pain medication in the last year	Higher ACE count was associated with greater odds of opioid prescription (logistic regression, OR 1.10, 95% CI 1.01–1.20).
Williams 2021	Adults with a history of at least one type of interpersonal trauma (intimate partner violence, sexual assault, and/or ACEs) recruited through public advertising	235	ACEs	Prescription opioid use	Self-completed online questionnaire asking if they had received a prescription for pain medication in the last year	ACE count was not associated with prescription opioid use (logistic regression, OR 1.07, 95% CI 0.94–1.23).
Wuest 2007	Women's Health Effects Study (WHES): English speaking women who had left an abusive partner in the preceding 3–36 months and who had a positive Abuse Assessment Screen	309	Abused as a child	Prescription pain medication use	Structured interview asking about use of prescription pain medications in the last month	A history of child abuse was associated with higher prescription pain medication use (χ^2^, *P*=0.02).
You 2019	Adults attending a university	3073	Childhood adversity	Prescription pain medication use	Self-completed online questionnaire asking to report current medications	Early traumatic inventory self-report score was not associated with prescription pain medication use (logistic regression, full result not provided, *P*>0.075).

### Outcome: analgesic side-effects

Four studies (involving 3491 participants) investigated analgesic medication side-effects (Table 2).^73^, ^74^, ^75^, ^76^ The largest study (*n*=3118) examined the most side-effects and found that people with a history of abuse were more likely to report ‘any side-effect’ and more likely to report 10 out of the 15 individual side-effects than those without a history of abuse.^76^ The side-effects considered included some that are often associated with opioid and gabapentinoid use (such as constipation, drowsiness, and nausea).^76^ However, Bottiroli and colleagues^73^ reported no influence of childhood trauma history (i.e. abuse and neglect) on the persistence of medication overuse-associated headache. In a double-blind placebo-controlled randomised trial by Carlyle and colleagues,^74^ after an intramuscular injection of morphine, participants with a history of childhood trauma reported higher ratings for the pleasurable side-effects (such as ‘feeling high’ and ‘liking drug effects’) and lower ratings for the disagreeable side-effects (such as ‘disliking drug effects’) than those with no such history. These findings were not replicated in a hospital-based study by the same research group, where day-case surgery patients were given an opioid infusion before general anaesthesia and asked similar questions.^75^

**Table 2:** Impact of adverse childhood experiences on analgesic side-effects. CI, confidence interval; NR, not reported; OR, odds ratio.

Author and yr	Participant population	Sample size	Exposure	Outcome	Outcome tool	Relationship
Bottiroli 2019	Adults with chronic migraine and medication overuse headache attending an inpatient detoxification clinic	166	Childhood trauma	Persistence of analgesia overuse 2 months after an analgesia detoxification programme Persistence of chronic headache 2 months after an analgesia detoxification programme	Clinical assessment by a neurologist	Number of emotional trauma events was associated with persistence of analgesia overuse at 2 months (logistic regression, OR 11.10, 95% CI 1.15–106.84). Total number of trauma events, number of physical trauma events, and sexual abuse history were not associated with the persistence of analgesia overuse at 2 months (logistic regression; data not reported). Total number of trauma events, number of emotional trauma events, number of physical trauma events, and sexual abuse history were not associated with the persistence of chronic headache at 2 months (logistic regression; data not reported).
Carlyle 2021	Adults recruited through ‘ … convenience and snowball sampling via participant databases, poster advertisements, and word of mouth … ’	52	Childhood trauma	Feeling the drug effects Feeling high Liking drug effects Wanting more of the drug Disliking drug effects	Self-completed questionnaire with questions from the Drug Effect Questionnaire, completed at baseline and then 15, 30, 45, 60, 90, 120, and 150 min after the administration of intramuscular morphine (0.15 mg/kg)	No significant difference between high trauma and no trauma groups for ‘feeling the drug effects’ (mixed-effects random intercept models, *P*>0.284). Compared with no trauma group, the high trauma group had higher ratings for ‘feeling high’ at 30 min after morphine administration (mixed-effects random intercept models, *P*=0.047), ‘liking drug effects’ at all time points (mixed-effects random intercept models, *P*<0.010), and ‘wanting more of the drug’ at all time points (mixed-effects random intercept models, *P*<0.001). Compared with high trauma group, the low trauma group had higher ratings for ‘disliking drug effects’ at 90 and 150 min after morphine administration (mixed-effects random intercept models, *P*=0.004 and *P*<0.001, respectively).
Carlyle 2023	Healthy adults (American Society of Anaesthesiologists grade 1–2) attending a day-case surgery unit	155	Childhood adversity	Post-opioid liking Post-opioid feeling good Post-opioid feeling high Post-opioid disliking Post-opioid feeling anxious	Structured interview using questions from the Drug Effect Questionnaire after the start of a remifentanil or oxycodone intravenous infusion, 5 min before a general anaesthetic	Higher total childhood trauma questionnaire score was associated with lower post-opioid liking (linear regression, β=–0.06, 95% CI –0.11 to –0.01). Total childhood trauma questionnaire score was not associated with post-opioid feeling good (linear regression, β=0.01, 95% CI –0.03 to 0.04), post-opioid feeling high (linear regression, β=–0.01, 95% CI –0.05 to 0.04), post-opioid disliking (linear regression, β=0.01, 95% CI –0.04 to 0.06), or post-opioid feeling anxious (linear regression, β=0.03, 95% CI –0.02 to 0.07).
Pierce 2020	Adults attending an outpatient pain clinic	3118	Child abuse	Any side-effect Drowsiness Constipation Fatigue Dizziness Nausea or vomiting Confusion Itchiness Other stomach/bowel upset Mental impairment Diarrhoea Swelling of hands and/or feet Difficulty urinating Heart palpitations Shortness of breath Hallucinations	NR	Compared with people with no abuse history, people with an abuse history reported higher proportions of any side-effect (χ^2^, *P*<0.001), drowsiness (χ^2^, *P*<0.001), constipation (χ^2^, *P*<0.001), fatigue (χ^2^, *P*<0.001), nausea or vomiting (χ^2^, *P*<0.001), confusion (χ^2^, *P*<0.001), itchiness (χ^2^, *P*=0.002), other stomach/bowel upset (χ^2^, *P*<0.001), mental impairment (χ^2^, *P*<0.001), diarrhoea (χ^2^, *P*<0.001), and heart palpitations (χ^2^, *P*<0.001). There was no difference in reporting of dizziness (χ^2^, *P*=0.007 [Bonferonni-corrected threshold of 0.003]), swelling of hands and/or feet (χ^2^, *P*=0.028 [Bonferonni-corrected threshold of 0.003]), difficulty urinating (χ^2^, *P*=0.049 [Bonferonni-corrected threshold of 0.003]), shortness of breath (χ^2^, *P*=0.103), or hallucinations (χ^2^, *P*=0.076).

### Outcome: substance misuse

Forty-five studies (involving 104 550 participants) investigated outcomes relating to substance misuse (Table 3).^69^^,^^70^^,^^77^, ^78^, ^79^, ^80^, ^81^, ^82^, ^83^, ^84^, ^85^, ^86^, ^87^, ^88^, ^89^, ^90^, ^91^, ^92^, ^93^, ^94^, ^95^, ^96^, ^97^, ^98^, ^99^, ^100^, ^101^, ^102^, ^103^, ^104^, ^105^, ^106^, ^107^, ^108^, ^109^, ^110^, ^111^, ^112^, ^113^, ^114^, ^115^, ^116^, ^117^, ^118^, ^119^ Of these, 44 studies examined opioids, and six examined sedatives.

**Table 3:** Impact of adverse childhood experiences on substance misuse. ACE, adverse childhood experience; CI, confidence interval; HR, hazard ratio; OR, odds ratio.

Author and yr	Participant population	Sample size	Exposure	Outcome	Outcome tool	Relationship
Afifi 2012	National Epidemiological Study of Alcohol and Related Conditions (NESARC), wave 2: a nationwide nationally representative household survey of adults	34 653	Childhood maltreatment	Sedative substance use disorder Opioid (unspecified) substance use disorder Heroin substance use disorder	Structured interview using questions from the Alcohol Use Disorder and Associated Disabilities Interview Schedule–DSM IV (AUDADIS-IV)	In males and females, physical abuse, sexual abuse, emotional abuse, physical neglect, and emotional neglect were associated with sedative substance use disorder (logistic regression; full results not reported here). In males and females, physical abuse, sexual abuse, emotional abuse, physical neglect, and emotional neglect were associated with opioid substance use disorder (logistic regression; full results not reported here). In males and females, physical abuse, sexual abuse, emotional abuse, and emotional neglect were associated with heroin substance use disorder (logistic regression; full results not reported here). In females, but not in males, physical neglect was associated with heroin substance use disorder (logistic regression; full results not reported here).
Ararso 2021	National Longitudinal Study of Adolescent to Adult Health (Add Health), waves 1, 3, and 4: a nationally representative cohort of adolescents aged 12–19 yr recruited from schools and followed up for more than 20 yr	12 288	Child abuse and homelessness	Prescription opioid misuse	Structured interview asking about prescription opioid use	At wave 3, compared with those with no history of abuse or homelessness, participants with abuse only (logistic regression, OR 1.41, 95% CI 1.17–1.69), homelessness only (logistic regression, OR 1.50, 95% CI 1.00–2.27), and abuse + homelessness (logistic regression, OR 3.23, 95% CI 2.11–4.93) had a higher odds of prescription opioid misuse. At wave 4, compared with those with no history of abuse or homelessness, participants with abuse only had a higher odds of prescription opioid misuse (logistic regression, OR 1.67, 95% CI 1.23–2.27). At wave 4, compared with those with no history of abuse or homelessness, participants with homelessness only (logistic regression, OR 1.58, 95% CI 0.84–2.94) and abuse + homelessness (logistic regression, OR 1.91, 95% CI 0.84–4.32) had no difference in the odds of prescription opioid misuse.
Austin 2018b	National Longitudinal Study of Adolescent to Adult Health (Add Health), waves 1 and 3: a nationally-representative cohort of adolescents aged 12–19 yr recruited from schools and followed up for more than 20 yr	14 322	Childhood abuse and neglect	Prescription opioid misuse	Structured interview asking about prescription opioid use since wave 1	Childhood abuse and neglect was associated with prescription opioid misuse (structural equation modelling, β=0.232, SE 0.022, *P*<0.001).
Browne 1998	Adults attending the outpatient National Drug Treatment Centre	52	Sexual abuse and physical abuse	Age of first opiate use Duration of opiate use	Semistructured interview	Sexual abuse was associated with a younger age of first opiate use (*t*-test, *P*=0.01). Sexual abuse was not associated with duration of opiate use (*t*-test; data not reported). Physical abuse was not associated with age of first opiate abuse or duration of opiate use (*t*-test; data not reported).
Carr 2023	Adults with opioid use disorder attending an outpatient addiction treatment centre	171	Childhood adversity	Opioid addiction severity	Self-completed questionnaire using the Recognizing Addictive Disorder (RAD) score	Higher ACE count was associated with higher opioid addiction severity score (linear regression, β=1.70, 95% CI 0.26–3.13).
Conroy 2009	Cases: adults attending opioid pharmacotherapy clinics Controls: adults recruited through public advertising	Cases: 967 Controls: 346	Childhood maltreatment	Opioid-dependence (unspecified)	Cases determined through enrolment in an opioid pharmacotherapy programme	In males, physical abuse was associated with being a case (logistic regression, OR 1.6, 95% CI 1.0–2.4). In males, sexual abuse (logistic regression, OR 0.7, 95% CI 0.5–1.1), emotional abuse (logistic regression, OR 1.2, 95% CI 0.8–1.9), and neglect (logistic regression, OR 1.2, 95% CI 0.8–1.8) were not associated with being a case. In females, physical abuse (logistic regression, OR 1.1, 95% CI 0.7–1.7), sexual abuse (logistic regression, OR 1.2, 95% CI 0.8–2.0), emotional abuse (logistic regression, OR 0.9, 95% CI 0.6–1.4), and neglect (logistic regression, OR 0.7, 95% CI 0.4–1.3) were not associated with being a case.
Davis 2022	Adolescents recruited from 16 middle schools and followed up annually for 12 yr, waves 8–12	2880	Victimisation	Latency to opioid misuse (prescription and illicit)	Self-completed online questionnaire with questions on past-year heroin use and past-year prescription narcotic medication use	Compared with the ‘low all’ ACE class, all classes were associated with a shorter latency to opioid misuse: ‘sexual abuse and indirect violence + high trauma characteristics’ (discrete time survival mixture analysis, HR 1.98, 95% CI 1.09–3.63), ‘high all + high trauma characteristics’ (discrete time survival mixture analysis, HR 2.00, 95% CI 1.32–3.03), and ‘chronic emotional abuse + trusted perpetrator’ (discrete time survival mixture analysis, HR 2.02, 95% CI 1.42–2.87).
Derefinko 2019	Adults attending an outpatient opioid use disorder clinic	87	ACEs	Opioid relapse	Positive result in patient self-report, urine drug screen, or prescription drug database result	Higher ACE count was associated with greater odds of opioid relapse (logistic regression, OR 1.17, 95% CI 1.05–1.30).
Dunn 2022	Adults with a history of heroin or prescription opioid use recruited online through Amazon Mechanical Turk	310	Early life trauma	Opioid use disorder (prescription and illicit) Opioid use disorder severity (prescription and illicit) Opioid withdrawal severity	Self-completed online questionnaire using questions from the DSM-V checklist for opioid use disorder	Total trauma score (*t*-test, *P*<0.001), general trauma score (*t*-test, *P*<0.001), physical trauma score (*t*-test, *P*=0.013), emotional trauma score (*t*-test, *P*=0.015), and sexual trauma score (*t*-test, *P*<0.001) were associated with opioid use disorder. Total trauma score (χ^2^, *P*<0.001), general trauma score (χ^2^, *P*<0.001), physical trauma score (χ^2^, *P*<0.001), emotional trauma score (χ^2^, *P*=0.002), and sexual trauma score (χ^2^, *P*<0.001) were associated with opioid use disorder severity. Total trauma score (Pearson's correlation, *r*=0.240, *P*<0.001), general trauma score (Pearson's correlation, *r*=0.238, *P*<0.001), physical trauma score (Pearson's correlation, *r*=0.151, *P*=0.008), emotional trauma score (Pearson's correlation, *r*=0.172, *P*=0.002), and sexual trauma score (Pearson's correlation, *r*=0.240, *P*=0.003) were associated with opioid withdrawal severity.
Eaves 2021	Adults incarcerated at a county detention facility	96	ACEs	Recent heroin use Recent other opiate use	Self-completed questionnaire asking about heroin/opiate use in the last 30 days before admission to jail	ACE count was associated with recent other opiate use (logistic regression, OR 1.26, 95% CI 1.00–1.61). ACE count was not associated with recent heroin use (logistic regression, OR 1.17, 95% CI 0.95–1.44).
Elhammady 2014	Cases: adults with opioid dependence syndrome, heroin dependence, or methadone prescription attending an outpatient addiction clinic Controls: friends and family members of cases	Cases: 120 Controls: 100	Child sexual abuse, child physical abuse, and parental history of drug/alcohol misuse	Severity of opioid dependence	Semistructured interview using the Severity of Dependence Scale and the Leeds Dependence Questionnaire	Sexual abuse (Pearson's correlation, *r*=0.185, *P*=0.043), physical abuse (Pearson's correlation, *r*=0.306, *P*=0.001), and parent drug/alcohol misuse (Pearson's correlation, *r*=0.245, *P*=0.007) were associated with Severity of Dependence Scale score. Sexual abuse (Pearson's correlation, r=0.180, p=0.049), physical abuse (Pearson's correlation, r=0.231, p=0.011), and parent drug/alcohol misuse (Pearson's correlation, *r*=0.285, *P*=0.002) were associated with Leeds Dependence Questionnaire score.
Fortson 2021	Adults attending a university	1402	ACEs	Risk for opioid misuse	Self-completed online questionnaire using questions from the Screener and Opioid Assessment for Patients with Pain (SOAPP)	ACE count was associated with greater odds of being high risk for opioid misuse (logistic regression; 0 ACEs: referent; 1–3 ACEs: OR 1.96, 95% CI 1.46–2.65; >4 ACEs: OR 2.93, 95% CI 1.95–4.39).
Fuss 2023	Vape shop Advertising, Place characteristics, and Effects Surveillance study (VAPES): adults aged 18–34 yr recruited online through Facebook and Reddit and followed up for 1 yr	2975	ACEs	Lifetime opioid use	Self-completed online questionnaire asking about lifetime opioid use, with participants categorised into (1) no opioid use, (2) prescription opioid use, (3) nonmedical prescription opioid use, and (4) heroin use groups	Those in the no-opioid group had a lower ACE count than those in the prescription opioid group (logistic regression, OR 0.92, 95% CI 0.87–0.97). Those in the heroin group had a higher ACE count than those in the prescription opioid group (logistic regression, OR 1.24, 95% CI 1.11–1.39). There was no difference in ACE counts between the nonmedical prescription opioid group and the prescription opioid group (logistic regression, OR 1.03, 95% CI 0.94–1.14).
Garami 2019	Cases: adults with a history of opiate addiction attending an opioid treatment programme. Controls: adults recruited via word of mouth from the community	Cases: 36 Controls: 33	Childhood trauma	Opiate addiction (unspecified)	Likelihood of being a case (i.e. having a history of opiate addiction and being treated at the clinic)	Higher childhood trauma questionnaire total score was associated with being a case (logistic regression, OR 1.11, 95% CI 1.04–1.18).
Garland 2019	Women with chronic pain and regular opioid analgesic use in the last 90 days attending primary care and specialty pain clinics	36	ACEs	Opioid use disorder severity (prescription) Negative emotional cue-elicited opioid craving	Structured interview using the Mini-International Neuropsychiatric Interview (MINI), followed by an experimental procedure in which participants were asked to rate opioid craving before and after exposure to negative affective images	ACE count was associated with opioid use disorder severity (linear regression, β=0.30, no 95% CI given, *P*=0.04). ACE count was associated with cue-elicited craving scores (linear regression, β=0.53, no 95% CI given, *P*=0.002).
Guarino 2021	Adults aged 18–29 yr with prescription opioid or heroin use in the preceding 30 days	539	ACEs	Age at initiation of nonmedical prescription opioid use Age at initiation of snorting of nonmedical prescription opioids Age at initiation of injection of nonmedical prescription opioids Age at initiation of heroin use Age at initiation of injection of heroin	Structured interview asking about age of initiation of various opioid use behaviours, with younger age defined as lowest quartile and older age was defined as highest quartile	Higher ACE count was associated with younger age at initiation of nonmedical prescription opioid use (logistic regression, OR 1.23, 95% CI 1.12–1.43), younger age at initiation of snorting of non-medication prescription opioids (logistic regression, OR 1.16, 95% CI 1.05–1.28), younger age at initiation of heroin use (logistic regression, OR 1.17, 95% CI 1.03–1.32), and younger age at initiation of injection of heroin (logistic regression, OR 1.13, 95% CI 1.02–1.25). ACE count was not associated with younger age at initiation of injection of nonmedication prescription opioids (logistic regression, OR 1.12, 95% CI 0.97–1.30).
Heffernan 2000	Adults attending an inpatient psychiatric hospital	763	Childhood abuse	Opiate use	Structured interview asking about opiate use	Childhood physical and/or sexual abuse was associated with opiate use (logistic regression, OR 2.68, 95% CI 2.27–3.10).
Khoury 2010	Adults attending outpatient general medical and OB/GYN clinics	587	Childhood traumatic experiences	Lifetime heroin/opiate use Past 30 days heroin/opiate use	Structured interview using the Kreek–McHugh–Schluger–Kellogg scale	Physical abuse score was associated with lifetime heroin/opiate use (Pearson's correlation, *r*=0.123, *P*<0.01) and past 30 days heroin/opiate use (Pearson's correlation, *r*=0.251, *P*<0.001). Sexual abuse score was not associated with lifetime heroin/opiate use (Pearson's correlation, *r*=0.038, *P*>0.01) or past 30 days heroin/opiate use (Pearson's correlation, *r*=–0.011, *P*>0.01). Emotional abuse score was not associated with lifetime heroin/opiate use (Pearson's correlation, *r*=0.045, *P*>0.01) or past 30 days heroin/opiate use (Pearson's correlation, *r*=0.144, *P*>0.01).
Kors 2022	Pregnant women (second trimester or later) attending a high-risk pregnancy clinic	93	Childhood maltreatment	Opioid use during pregnancy (prescription and illicit)	Urine analysis for prescribed or nonprescribed opioids	Sexual abuse was associated with opioid use during pregnancy (logistic regression, OR 3.17, 95% CI not provided, *P*=0.03). Physical abuse (logistic regression, OR 1.21, 95% CI not provided, *P*=0.73), neglect (logistic regression, OR 1.24, 95% CI not provided, *P*=0.44), and emotional abuse (logistic regression, OR 1.10, 95% CI not provided, *P*=0.72) were not associated with opioid use during pregnancy.
Kumar 2016	Adults attending an outpatient buprenorphine treatment programme	113	Childhood trauma	Dropping out of a buprenorphine treatment programme Phase advancement in a buprenorphine treatment programme	Absence >8 days during the first 90 days of the programme Advancement from weekly to biweekly visits during the first 90 days of the programme	Moderate/severe physical neglect score (logistic regression, OR 4.84, 95% CI 1.33–17.65) and moderate/severe emotional neglect score (logistic regression, OR 8.27, 95% CI 1.51–45.39) were associated with greater odds of dropping out of the buprenorphine treatment programme. Moderate/severe physical abuse, emotional abuse, and sexual abuse were not associated with dropping out of the buprenorphine treatment program (χ^2^; data not provided). Moderate/severe physical abuse, emotional abuse, sexual abuse, physical neglect, and emotional neglect were not associated with phase advancement in the buprenorphine treatment (χ^2^; data not provided).
Larance 2018	Adults with a history of heroin dependence receiving opioid agonist treatment	1149	Childhood maltreatment	Transition from heroin use to dependence Transition from heroin dependence to treatment seeking	Structured interview using questions from the Semi-Structured Assessment of the Genetics of Alcoholism–Australia (SSAGA-OZ)	Childhood maltreatment count was associated with higher odds of transitioning from heroin use to dependence (multivariate discrete-time survival analysis, OR 1.11, 95% CI 1.04–1.18). Childhood maltreatment count was not associated with higher odds of transitioning from heroin dependence to treatment seeking (multivariate discrete-time survival analysis, OR 1.01, 95% CI 0.95–1.07).
Lynskey 2006	The Australian Twin Study: twins recruited from schools in Australia and followed up through adulthood	6265	Childhood sexual abuse; childhood physical abuse	Opioid + sedative abuse/dependence	Structured telephone interview using the modified Semi-Structured Assessment for the Genetics of Alcoholism (SSAGA) tool, with subsequent latent class analysis identifying five substance use classes	Compared with the ‘low use’ class, the ‘opioid + sedative’ class had higher odds of physical abuse (logistic regression, OR 3.8, 95% CI 2.8–5.3) and sexual abuse (logistic regression, OR 4.0, 95% CI 2.6–6.0).
Martin 2023	Adults with substance use disorder recruited via media advertising	565	ACEs	Opioid use	Structured interview with questions on history of opioid use.	Compared with people with tobacco use only, people with opioid use had higher odds of reporting high ACE counts (ordinal logistic regression, OR 2.21, 95% CI 1.27–3.86). Compared with people with tobacco use only, people with opioid use had higher odds of reporting household dysfunction (logistic regression, OR 2.67, 95% CI 1.30–5.49). There was no association between people with tobacco use only and people with opioid use when reporting emotional/physical abuse (logistic regression, OR 1.46, 95% CI 0.79–2.69), sexual abuse (logistic regression, OR 1.54, 95% CI 0.74–3.20), or neglect (logistic regression, OR 1.89, 95% CI 0.95–3.75).
McDonagh 2023	Adults attending substance use services	104	ACEs	Age of first opiate use	Structured interview using the Opiate Treatment Index (OTI).	Higher ACE count was associated with a younger age of first opiate use (Spearman correlation, *r*=–0.278, *P*<0.01).
Meadows 2023	Adults recruited through public advertising	107	ACEs	Age of initiation of nonmedical prescription opioid use Age of initiation of non-medical prescription benzodiazepine use	Self-completed questionnaire with a drug history screening tool followed by an ‘ … an in-depth questionnaire to evaluate use patterns, including age of initiation … ‘.	Higher ACE count was associated with a younger age of initiation of nonmedication prescription opioid use (Cox proportional hazard regression, OR 1.22, 95% CI 1.05–1.43). Higher ACE count was not associated with a younger age of initiation of nonmedical prescription benzodiazepine Cox proportional hazard regression, OR 1.03, 95% CI 0.90–1.19).
Merrick 2020	Behavioural Risk Factor Surveillance System (BRFSS), Montana and Florida populations: adults responding to a telephone survey	Montana: 8726 Florida: 27 545	ACEs	Prescription opioid misuse	Montana: self-completed questionnaire asking about use of prescription opioids at a higher frequency/dose than prescribed or without a prescription. Florida: self-completed questionnaire asking about use of prescription opioids without a prescription or for the experience/feeling	Montana: Higher ACE count was associated with higher odds of using prescribed opioids at a higher frequency/dose than prescribed (logistic regression; 0 ACEs: referent; 1–2 ACEs: OR 2.82, 95% CI 1.06–7.46; ≥3 ACEs: OR 4.73, 95% CI 1.79–12.51) and using opioids without a prescription (logistic regression; 0 ACEs: referent; 1–2 ACEs: OR 3.53, 95% CI 2.11–5.92; ≥3 ACEs: OR 7.13, 95% CI 4.32–11.77). Florida: Higher ACE count was associated with higher odds of using prescription opioids without a prescription or for the experience/feeling (logistic regression; 0 ACEs: referent; 1–2 ACEs: OR 1.55, 95% CI 0.98–2.45; ≥3 ACEs: OR 3.08, 95% CI 1.65–5.75).
Mirhashem 2017	Adults with a history of opioid use >1 yr	84	Childhood maltreatment	Substance related problems	Self-completed questionnaire using the Short Inventory of Problems-Revised (SIPS-R) tool, which measures the negative effects of drug use	Physical neglect score (Pearson's correlation, *r*=0.06, *P*>0.05), emotional neglect score (Pearson's correlation, *r*=–0.02, *P*>0.05), sexual abuse score (Pearson's correlation, *r*=0.03, *P*>0.05), physical abuse score (Pearson's correlation, *r*=0.05, *P*>0.05), and emotional abuse score (Pearson's correlation, *r*=0.17, *P*>0.05) were not associated with higher substance related problems.
Myers 2014	National Epidemiological Study of Alcohol and Related Conditions (NESARC), wave 2: a nationwide nationally representative household survey of adults	34 653	Childhood adversity	Disordered opiate use in the last 12 months (unspecified)	Structured interview using questions from the Alcohol Use Disorder and Associated Disabilities Interview Schedule–DSM IV (AUDADIS-IV)	Childhood adversity count was associated with greater odds of disordered opiate use in the last 12 months (logistic regression; 0 ACE: referent; 1–2 ACE: OR 15.8, 95% CI 1.72–145.5; ≥3 ACE: OR 19.5, 95% 1.82–208.1).
Naqavi 2011	Cases: opiate dependent adults attending drug treatment centres. Controls: adults who visited neighbourhood clinics for reasons other than addiction treatment	Cases: 212 Controls: 216	Childhood maltreatment	Opiate dependency	Likelihood of being a case (i.e. having a history of opiate dependency and being treated at the clinic).	Emotional abuse (logistic regression, OR 5.06, 95% CI 2.30–11.18), sexual abuse (logistic regression, OR 1.89, 95% CI 1.04–3.43), and physical neglect (logistic regression, OR 1.96, 95% CI 1.21–3.18) were associated with being a case. Physical abuse and emotional neglect were not associated with being a case (logistic regression; data not provided).
Nelson 2006	The Australian Twin Study: twins recruited from schools in Australia and followed up through adulthood	6050	Childhood sexual abuse	Opioid abuse/dependence Sedative abuse/dependence	Structured telephone interview using the modified Semi-Structured Assessment for the Genetics of Alcoholism (SSAGA) tool	Sexual abuse was associated with a high risk for opioid abuse/dependence (Cox proportional hazard regression, HR 2.60, 95% CI 1.60–4.22) and sedative abuse/dependence (Cox proportional hazard regression, HR 2.61, 95% CI 1.43–4.78). When looking at same-sex twins with different sexual abuse histories, sexual abuse was associated with a high risk of opioid abuse/dependence (conditional logistic regression, OR 6.50, 95% CI 1.47–28.80). When looking at same-sex twins with different sexual abuse histories, sexual abuse was not associated with sedative abuse/dependence.
Onu 2021	Adults attending a university, recruited from local student accommodation and hostels	301	ACEs	Tramadol abuse	Self-completed questionnaire using questions from the Tramadol Abuse Scale	Higher ACE count was associated with a higher tramadol abuse score (Pearson's correlation, *r*=0.33, *P*<0.001).
Pakdaman 2021	Adults attending a university	3899	ACEs	Prescription opioid misuse Prescription sedative misuse	Self-completed online questionnaire asking about use of prescription drugs without a prescription (response classes were antidepressants, painkillers, sedatives, and stimulants)	Higher ACE count was associated with higher odds of prescription opiate misuse (logistic regression, OR 1.21, 95% CI 1.13–1.30) and prescription sedative misuse (logistic regression, OR 1.33, 95% CI 1.22–1.44).
Quinn 2016	National Longitudinal Study of Adolescent to Adult Health (Add Health), waves 3, and 4: a nationally representative cohort of adolescents aged 12–19 yr recruited from schools and followed up for more than 20 yr	12 288	Childhood trauma	Prescription pain reliever misuse	Structured interview asking about prescription pain reliever use	At wave 3, ACE count was associated with prescription pain reliever misuse (logistic regression; 0 ACEs: referent; 1 ACE: OR 1.34, 95% CI 1.14–1.58; 2 ACEs: OR 1.58, 95% CI 1.25–1.99; 3 ACEs: OR 1.70, 95% CI 1.29–2.25; 4 ACEs: OR 2.17, 95% CI 1.49–3.15; ≥5 ACEs: OR 1.79, 95% CI 1.05–3.07). At wave 4, ACE count was associated with prescription pain reliever misuse (logistic regression; 0 ACEs: referent; 1 ACE: OR 1.46, 95% CI 1.12–1.91; 2 ACEs: OR 1.71, 95% CI 1.23–2.36; 3 ACEs: OR 2.16, 95% CI 1.43–3.26; 4 ACEs: OR 2.70, 95% CI 1.62–4.52; ≥5 ACEs: OR 3.09, 95% CI 1.52–6.30).
Santo Jr 2022	Pain and Opioids in Treatment (POINT) study: adults prescribed regulated opioids for chronic noncancer pain recruited from community pharmacies	1514	Childhood trauma	Opioid use disorder (prescription and illicit)	Structured interview following the Composite International Diagnostic Interview (CIDI) v3 asking about opioid use in the past 12 months	Compared with the ‘low exposure’ ACE class, the ‘emotional and sexual abuse’ class (logistic regression, OR 1.75, 95% CI 1.25–2.34) and the ‘high all’ class (logistic regression, OR 2.75, 95% CI 2.04–3.70) were associated with opioid use disorder.
Sartor 2014	Adults with opioid dependence attending for an alternative research study	3513	Childhood risk factors	Transition time from first opioid use to opioid dependence	Structured interview based on questions covering ‘ … age at first use … ’ and ‘ … age at dependence onset, defined as the age at which full dependence criteria were met … ’	Severe physical abuse was associated with quicker transition time (ordinal logistic regression, OR 1.50, 95% CI 1.17–1.91). Other ACEs were not associated with quicker transition (data not reported).
Stein 2017	Adults attending an inpatient opioid detoxification programme	457	ACEs	Age of initiating opioid use Recent intravenous drug use	Structured interview asking about lifetime opioid use and intravenous use within the last month	Higher ACE count was associated with younger age of opioid initiation (linear regression, β=–0.50, 95% CI –0.70 to –0.29). Higher ACE count was associated with greater odds of recent intravenous drug use (logistic regression, OR 1.11, 95% CI 1.02–1.20).
Tang 2020	National Epidemiological Study of Alcohol and Related Conditions (NESARC), wave 3: a nationwide nationally representative household survey of adults	36 309	ACEs	Past-year prescription opioid misuse Lifetime prescription opioid misuse Early-onset status of prescription opioid misuse Past-year opioid use disorder Lifetime opioid use disorder	Structured interview with questions on painkiller use and from the Alcohol Use Disorder and Associated Disabilities Interview Schedule-5 (AUDADIS-5), with ‘early onset’ defined as ≤17	Higher ACE count was associated with past-year prescription opioid misuse (logistic regression, OR 1.03, 95% CI 1.00–1.06), lifetime prescription opioid misuse (logistic regression, OR 1.04, 95% CI 1.02–1.06), early-onset status of prescription opioid misuse (logistic regression, OR 1.07, 95% CI 1.03–1.10), and lifetime opioid use disorder (logistic regression, OR 1.06, 95% CI 1.02–1.10). ACE count was not associated with past-year opioid use disorder (logistic regression, OR 1.05, 95% CI 0.99–1.10).
Taplin 2014	Adults with a history of opioid injection who had previously participated in the North American Opiate Medication Initiative (NAOMI)	87	Childhood trauma	Age of first injection of opioids	Structured interview using ‘ … the Family History section of the European Addiction Severity Index (EuropASI) … ’	All forms of childhood trauma were associated with a younger age of first injection of opioids (regression models, unclear adjustments): emotional abuse, 1.19% decrease in age (95% CI 0.06–2.32); physical abuse, 1.44% decrease in age (95% CI 0.20–2.67); sexual abuse, 1.31% decrease in age (95% CI 0.33–2.29); emotional neglect, 1.69% decrease in age (95% CI 0.48–2.88); and physical neglect, 2.07% decrease in age (95% CI 0.64–3.48).
Thiesset 2023	Adults with a history of opioid use disorder identified through the University of Utah's health system's electronic data warehouse	124	ACEs	Acknowledging a history of opioid use disorder	Self-completed online questionnaire asking about opioid use disorder, including ‘ … a history of addiction … ’ and ‘ … a history of using opioids prescribed to another person … ’	Exposure to ≥4 ACEs was associated with acknowledging a history of opioid use disorder on the survey (χ^2^, *P*=0.04).
Tomassi 2017	Adults aged 18–54 yr with first presentation of psychosis attending community mental health centres	345	Childhood trauma	Lifetime use of heroin	Structured interview using the Cannabis Experiences Questionnaire (assesses cannabis, cocaine, and heroin)	Severe sexual abuse (logistic regression, OR 12.6, 95% CI 2.7–58.1) and severe physical abuse (logistic regression, OR 3.7, 95% CI 1.2–11.3) were associated with lifetime heroin use. There was no difference between the trauma and no-trauma groups and lifetime heroin use (χ^2^, *P*=0.74).
Vogel 2011	Adults with opioid dependence attending outpatient clinics	193	Traumatic childhood experiences	Prolonged benzodiazepine use Lifetime benzodiazepine use	Self-completed questionnaire asking questions on prolonged benzodiazepine use (>2 months) and any lifetime benzodiazepine use	Higher childhood trauma questionnaire score was associated with prolonged benzodiazepine use (logistic regression, OR 1.53, 95% CI 1.11–2.11). There was no association between childhood trauma questionnaire score and lifetime benzodiazepine use (Mann-Whitney-*U* test, *P*=0.21).
Wang 2021	National Epidemiological Study of Alcohol and Related Conditions (NESARC), wave 3: a nationwide nationally representative household survey of adults	33 613	ACEs	Prescription opioid misuse	Structured interview asking about nonmedical use of prescription opioids in the last 12 months	Higher ACE count was associated with greater odds of prescription opioid misuse in the last year (generalised structural equation modelling, OR 1.09, 95% CI 1.05–1.13).
Widom 2006	Children <12 yr with court-substantiated abuse/neglect between 1967 and 1971 and matched nonabused controls, followed up for more than 20 yr	892	Child abuse and/or neglect	Lifetime heroin use Past-year heroin use	Structured interview asking about drug use patterns, including lifetime and past-year use	Abuse/neglect status was not associated with lifetime heroin use (logistic regression, OR 1.29, 95% CI 0.67–2.50) or with past-year heroin use (logistic regression, OR 1.60, 95% CI 0.14–17.69).
Williams 2020	Adults with a history of at least one type of interpersonal trauma (intimate partner violence, sexual assault, or ACEs) recruited through public advertising	230	ACEs	Opioid misuse (prescription and illicit)	Self-completed online questionnaire with questions on past-year heroin use, past-year prescription pain medication use without a prescription, and the PROMIS1 Prescription Pain Medication Misuse 7a Scale	ACE count was not associated with opioid misuse (logistic regression, OR 1.10, 95% CI 0.99–1.22).
Williams 2021	Adults with a history of at least one type of interpersonal trauma (intimate partner violence, sexual assault, or ACEs) recruited through public advertising	235	ACEs	Opioid misuse (prescription and illicit)	Self-completed online questionnaire with questions on past-year heroin use, past-year prescription pain medication use without a prescription, and the PROMIS1 Prescription Pain Medication Misuse 7a Scale	Higher ACE count was associated with greater odds of opioid misuse (logistic regression, OR 1.32, 95% CI 1.16–1.49).

ACE exposure was associated with prescription opioid misuse in all seven of the included studies that examined this relationship (7/7 studies), illicit opioid misuse in 2/5 studies, and unspecified opioid misuse in 12/15 studies. A number of the 45 included studies reported outcomes relating to different aspects of opioid misuse development and progression. Compared with those who had experienced no/low ACE exposure, high ACE exposure was associated with a higher risk for opioid misuse in 1/1 study, an earlier age of first opioid misuse in 5/6 studies, an earlier age of first opioid injection in 2/2 studies, a shorter latency from opioid use to misuse in 2/3 studies, a higher severity of opioid use disorder in 4/4 studies, a higher severity of opioid withdrawal in 1/1 study, and a greater likelihood of opioid relapse in 1/1 study. Compared with no/low ACE exposure, high ACE exposure was not associated with the duration of opioid misuse in 1/1 study, and had an equivocal association with engagement within a rehabilitation programme in 1/1 study. Compared with lower ACE exposure, high ACE exposure was associated with sedative misuse in 4/5 studies but was not associated with an earlier age of first sedative use in 1/1 study.

### Outcome: lifetime drug overdose

Two studies (involving 658 participants) investigated lifetime incidence of drug (including opioid) overdose (Table 4).^112^^,^^120^ In both studies, a higher ACE count was associated with a greater likelihood of lifetime drug overdose.

**Table 4:** Impact of ACEs on lifetime drug overdose. ACE, adverse childhood experience; CI, confidence interval; OR, odds ratio; RR, relative risk.

Author and yr	Participant population	Sample size	Exposure	Outcome	Outcome tool	Relationship
El-Bassel 2019	Women participating in Project PACT (a couple-focused randomised clinical trial of an HIV prevention intervention for men undergoing community corrections and their female intimate partners) who reported lifetime use of illicit drugs	201	Childhood adversity	Lifetime overdose	Structured interview asking about lifetime overdose (defined as loss of consciousness) of heroin, opioid pain relievers, or tranquiliser	Higher childhood adversity count was associated with greater risk of lifetime overdose (generalised linear model, RR 1.3, 95% CI 1.1–1.6).
Stein 2017	Adults attending an inpatient opioid detoxification programme	457	ACEs	Lifetime overdose	Structured interview asking about lifetime overdose	Higher ACE count was associated with greater odds of lifetime overdose (logistic regression, OR 1.10, 95% CI 1.02–1.20).

### Outcome: endogenous pain signalling

Four studies (involving 353 participants) investigated endogenous pain signalling pathways (Table 5).^121^, ^122^, ^123^, ^124^ Zehetmeier and colleagues^124^ performed quantitative sensory testing and found no association between ACE exposure and pain thresholds in various sensory modalities. Hill and colleagues^122^ found no association between ACE exposure and μ-opioid receptor binding in two regions of the brain (nucleus accumbens and amygdala). Two studies looked at the hypothalamic–pituitary–adrenal (HPA) axis: Groh and colleagues^121^ showed that ACE exposure was associated with a lower baseline cortisol level and a slower decrease in cortisol following an intravenous opioid agonist (diamorphine), and Lovallo and colleagues^123^ found that ACE exposure was associated with a smaller cortisol increase after an oral opioid antagonist (naltrexone).

**Table 5:** Impact of adverse childhood experiences on endogenous pain signalling. ACTH, adrenocorticotropic hormone; anova, analysis of variance.

Author and yr	Participant population	Sample size	Exposure	Outcome	Outcome tool	Relationship
Groh 2020	Adults with opioid dependency attending a diamorphine maintenance treatment clinic	15	Childhood trauma	Cortisol response to diamorphine ACTH response to diamorphine	Plasma cortisol/ACTH measured at –15, 15, 60, 180, and 300 min relative to intravenous diamorphine injection	Compared with the mild trauma group, the severe trauma group had a lower baseline cortisol (anova, *F*[1.140]=39.93, *P*<0.001). Compared with the mild trauma group, the severe trauma group had a slower decrease in cortisol following diamorphine injection (anova, *F*[1.6]=9.38, *P*=0.022). Trauma group status was not associated with baseline ACTH (results not provided) or the decrease in ACTH following diamorphine injection trauma (anova, *F*[1.6]=1.69, *P*=0.242).
Hill 2022	Adults recruited through public advertising	75	Childhood maltreatment	Mu opioid receptor density in the nucleus accumbens and amygdala	Binding of radiolabelled opioid (11C-carfentanil) administered by intravenous infusion to the nucleus accumbens and amygdala, assessed by MRI and PET scan	Childhood trauma questionnaire score was not associated with Mu opioid receptor binding (linear mixed-effects model, *F*=3.28. *P*=0.074).
Lovallo 2018	Oklahoma Family Health Patterns Project: healthy women aged 18–30 yr	72	Early-life adversity	Cortisol response to naltrexone Subjective distress and activation scores in response to naltrexone	Salivary cortisol measured every 30 mins for 180 mins after oral naltrexone or placebo ingestion Visual analogue scales on distress (five items) and activation (five items) completed every 60 min for 180 min after oral naltrexone or placebo ingestion	Higher early-life adversity score was associated with a smaller cortisol increase in response to oral naltrexone (anova, *F*=3.51, *P*=0.035). Higher early-life adversity score was associated with a smaller/negligible increase in distress score (anova, *F*=4.13, *P*=0.02). Early-life adversity score was not associated with activation score (anova, full results not provided, *P*>0.278).
Zehetmeier 2023	Pregnant women (third trimester) attending an inpatient obstetric clinic	191	Adverse childhood experiences	Quantitative sensory assessment (mechanical detection threshold, mechanical pain threshold, pressure pain threshold, and conditioned pain modulation)	Full description of sensory testing protocol not reported here	There was no association between trauma exposure and quantitative sensory assessment parameters: mechanical detection threshold (anova, *F*=0.20, *P*=0.654), mechanical pain threshold (anova, *F*=0.01, *P*=0.911), pressure pain threshold (anova, *F*=0.18, *P*=0.671), and conditioned pain modulation (anova, *F*=0.03, *P*=0.861).

### Outcome: other analgesia-related outcomes

Two studies involved outcomes that did not fit into the above five categories (Table 6).^125^^,^^126^ Roy and colleagues^125^ reported that some types of ACE (emotional abuse, sexual abuse, and emotional neglect) were associated with attempted suicide in adults with opioid dependency. Smith and colleagues^126^ reported that ACE exposure was not associated with use of the recreational herbal agent, kratom, which contains constituents known to activate the μ-opioid receptor.^127^

**Table 6:** Impact of ACEs on other analgesia-related outcomes. ACE, adverse childhood experience; CI, confidence interval; OR, odds ratio.

Author and yr	Participant population	Sample size	Exposure	Outcome	Outcome tool	Relationship
Roy 2002	Adults with opiate dependency attending substance abuse clinics	246	Childhood trauma	Attempted suicide	‘ … a self-destructive act with some intent to end one's life that was not self-mutilatory in nature … ’	Emotional abuse: OR 8.90 (95% CI 1.28–68.50) Sexual abuse: OR 12.90 (95% CI 1.47–165.20) Emotional neglect: OR 5.70 (95% CI 1.17–28.70) No association for physical abuse or physical neglect (data not provided)
Smith 2022	Adults with a history of alcohol, prescription opioid, illicit opioid, kratom, or illicit stimulant use in the last 6 months, recruited through Amazon Mechanical Turk	1510	ACEs	Lifetime kratom use	Self-completed online questionnaire with a question about lifetime kratom use	In unadjusted analysis, ACE count was higher in the lifetime kratom use group than in the no kratom use group (*t*-test, Cohen's d=–0.34, *P*=0.001). In adjusted analysis, there was no significant association between total ACE count and lifetime kratom use (logistic regression, OR 0.94, 95% CI 0.89–1.03).

## Discussion

In this systematic review, we synthesised the literature on adverse childhood experiences and a range of analgesia-related outcomes, using data from 137 395 participants (66 studies). In general, high ACE exposure was associated with poorer outcomes than no/low ACE exposure. However, there were differences in the way that the included studies defined, measured, and analysed their key variables, so this synthesis should be interpreted with these limitations in mind.

When considering analgesic medication use, our results showed that compared with no/low ACE exposure, high ACE exposure was generally associated with greater use. No data were presented in the included studies to allow us to draw firm conclusions as to why this may be the case; it could reflect the higher prevalence of chronic pain in ACE-exposed individuals, a greater severity of chronic pain experienced, a greater perceived need for treatment, a greater risk of analgesic misuse, or some other factors altogether. We found it notable that no studies reported on whether ACEs influenced the benefits of analgesic medications (e.g. by affecting functional status or pain scores).

When considering analgesic medication harms, our results showed that compared with no/low ACE exposure, high ACE exposure was generally associated with greater likelihood of harm. The majority of the included studies, especially in the context of substance misuse, looked specifically at opioids, which is perhaps unsurprising given the high profile given to the opioid crisis in both the scientific and popular press.^33^, ^34^, ^35^^,^^128^ Opioid-related harms included a higher incidence of analgesic medication side-effects, greater risk and severity of substance misuse, greater risk of drug overdose, and greater risk of attempted suicide in those misusing opioids. The only other drug class to be represented was sedatives (e.g. benzodiazepines), which are not formal analgesics and whose use in the management of chronic pain is generally not recommended.^129^ We included them in our search strategy as they are sometimes prescribed for pain, although the association between ACE exposure and sedative misuse summarised here may provide another reason to caution against their regular use. We found it notable that other analgesics with an established risk of harm were not investigated; for example, we found no studies that specifically investigated ACE exposure and gabapentinoid outcomes, which in recent years have been shown to have similar public health risks as opioids.^34^^,^^43^^,^^49^

The links between ACEs and opioid-related harms found in our review were concordant with similar reviews focussing specifically on ACEs and opioid use.^130^^,^^131^ Two of the included studies hinted at a possible mechanism for this relationship: high ACE exposure was associated with changes to the HPA axis, altering the stress hormone responses to opioid agonists and antagonists.^121^^,^^123^ This would be in line with existing evidence showing that people with ACE exposure have blunted cortisol reactivity to stressors^132^ and the general mechanisms proposed to contribute to the syndrome of ‘toxic stress’.^19^ However, the relationship between ACEs and the HPA axis is complicated, with a recent meta-analysis suggesting that ACE factors (e.g. type of adversity) and participant factors (e.g. racial background) may influence the strength and direction of the correlation.^133^ It is of note that one included study used an objective measure of pain sensation (quantitative sensory testing) but did not find an association between ACEs and pain thresholds in various sensory modalities.^124^ However, the population in this study was pregnant women in the third trimester, so it is unclear how generalisable this would be to the broader adult population. It is likely that the long-term consequences of ACEs occur through multiple pathways, although specific evidence relating ACEs to analgesia-related outcomes is currently lacking.

There are a number of strengths to our review. Firstly, we used a robust methodology, including a broad search string that made no assumptions about the type of analgesia-related outcome that might be discovered. As a result, we are confident that we have identified the relevant literature. Secondly, our findings are based on data from a large number of participants (*n*=137 395) from a range of contexts, including healthcare settings and dedicated research cohorts, which we believe will reflect a variety of real-world contexts.

However, there are also some limitations to the generalisability of our findings, which are predominantly attributed to the nature of the studies included in the review. Firstly, there was substantial heterogeneity in the definition and operationalisation of ACEs across the included studies, with the upper age threshold for childhood ranging from <12 to <19 yr and the number of included ACE types ranging from 1 to 27. This is an issue for any synthesis of ACE research, as there is no consistency or consensus opinion on what constitutes an ACE.^134^ Furthermore, our patient and public involvement group strongly highlighted the limitation of an assumption central to most ACEs research: that different ACEs in the same person or the same ACE in different people carry the same burden of stress. Ten included studies partially addressed this by incorporating measures of frequency or severity into an ACE score, but these were objective assessments that did not directly assess the subjective impact of an ACE. It also added an additional layer of heterogeneity that made comparisons between studies more difficult.

Secondly, the included studies used retrospective participant reports of ACE exposure and so were at risk of recall and reporting bias. This is the case for much of the research into ACEs, especially given the potential ethical issues arising from prospective ACE measurement without intervention. Two studies that compared prospective measurements of ACEs during childhood with retrospective recall of ACEs during adulthood found inconsistent results.^135^^,^^136^ Recall and reporting bias may also be influenced by later life experiences: our patient and public involvement group reported their perception that people exposed to ACEs who subsequently had supportive relationships seemed less likely to have poor long-term outcomes than those who experienced abusive relationships in adulthood. Such adult circumstances are rarely captured in ACE research and were not considered moderators in any of the studies included in this review.

Thirdly, there is undoubtedly a close relationship between ACEs and childhood socioeconomic status (SES),^137^ but for our review, it was not possible to separate the effects of the two variables on our outcomes owing to the limited reporting of childhood SES in the included studies. The studies that did acknowledge SES used measures from adulthood (e.g. income level and education level) that could have been influenced by ACEs in earlier life and therefore introduced another potential source of bias attributed to confounding (ROBINS-E domain 1; Supplementary Table S3). Furthermore, different measures of SES may produce different apparent effects; thus, it remains difficult to adequately isolate the impact of each concept.^138^

Fourthly, although a number of the included studies incorporated analysis of factors that may moderate the relationship between ACEs and analgesia-related outcomes, the heterogeneity of these variables across studies precluded any meaningful synthesis. Potential factors that were identified (all by just one study each) included the following: the presence of adolescent pain,^79^ perceived stress,^85^ any past or existing health condition,^88^ sociosexuality,^107^ a centralised pain phenotype,^76^ pain catastrophising,^76^ internalising symptoms (e.g. anxiety and depression),^113^ and externalising symptoms (e.g. aggression and delinquency).^113^ There are likely to be additional factors not described here that may also influence this relationship. For example, demographic factors (e.g. gender), co-morbid health conditions (especially mental health conditions such as posttraumatic stress disorder), and co-administration of multiple pharmacologic agents were not evaluated.

Our review aligns with previously published work reporting the impact of ACEs on long-term health outcomes^1^, ^2^, ^3^, ^4^, ^5^, ^6^, ^7^, ^8^, ^9^ and advances our knowledge on the specific field of ACEs and chronic pain. It lends additional weight to the benefit of adopting a trauma-informed model of care, in which the potential long-term impacts of negative experiences in childhood are acknowledged in the assessment and management of disease.^139^^,^^140^

Although widespread screening for ACEs is contro-versial,^141^, ^142^, ^143^, ^144^ assessment of ACEs in chronic pain settings may have benefits by identifying those at greater risk of analgesic harms—either in those who are newly starting medication or in those who are established on analgesics and who may benefit from a prescribing review. At the very least, it provides some context for management discussions between individuals living with chronic pain and healthcare professionals. However, we should keep in mind that the evidence about the impact of ACEs on the effectiveness of analgesics and the harms of non-opioid analgesics is still patchy at best.

Our review focused on the pharmacological management of chronic pain, but this is by no means the only management option available. Indeed, recent guidelines advocate for a move away from traditional analgesics (e.g. opioids) with deprescribing encouraged where appropriate.^145^ The impact of ACEs on the nonpharmacological management of chronic pain is also an area where more research is required, although a recent study found that ACE exposure did not influence the improvements in pain and functional status after an interdisciplinary pain rehabilitation programme.^146^ More broadly, the evidence for interventions to support people exposed to ACEs is mixed, with no clear consensus on the best approach.^147^^,^^148^ The reality is that each individual's experience of ACEs is different; therefore, they will likely require different levels and modalities of support.

In summary, this systematic review evaluated the literature on adverse childhood experiences and a range of analgesia-related outcomes. In general, higher adverse childhood experience exposure was associated with poorer outcomes than lower/no exposure. This included greater use of analgesic medication, greater incidence of analgesic medication side-effects, greater risk and severity of substance misuse, greater risk of drug overdose, and greater risk of attempted suicide in opioid dependency. Higher adverse childhood experience exposure was also associated with changes to the HPA axis, altering stress hormone responses to opioid agonists and antagonists. However, the evidence base is heterogenous, and there are still gaps in our understanding. Nevertheless, on the basis of current evidence, we believe that adopting trauma-informed practices in settings where adverse childhood experience exposures are common (e.g. the chronic pain clinic) is justified and may help to improve the management of chronic pain.

## Authors’ contributions

Study conception and design: DS, LC

Acquisition of data: DS, MK, KB

Analysis and interpretation of data: DS, MK, KB, BS, TH, LM, LC

Drafting of final manuscript: DS

Revision and critical appraisal of the manuscript: MK, KB, BS, TH, LM, LC

Final approval of the submitted manuscript: DS, MK, KB, BS, TH, LM, LC

## Declarations of interest

LC is a member of the *BJA Open* editorial board. DS, MK, KB, BS, TH, and LM declare that they have no competing interests.

## Funding

The authors are members of the Advanced Pain Discovery Platform (APDP) supported by 10.13039/100014013UK Research and Innovation (UKRI), Versus Arthritis, and Eli Lilly. DS is a fellow on the Multimorbidity Doctoral Training Programme for Health Professionals, which is supported by the Wellcome Trust (223499/Z/21/Z). MK is funded by The National Institute of Academic Anaesthesia (NIAA) (WKR0-2022-0028). BS and LC are supported by an APDP grant as part of the Partnership for Assessment and Investigation of Neuropathic Pain: Studies Tracking Outcomes, Risks and Mechanisms (PAINSTORM) consortium (MR/W002388/1). TH and LC are supported by an APDP grant as part of the Consortium Against Pain Inequality (MR/W002566/1). The funding bodies had no role in study design, data collection/analysis/interpretation, report writing, or the decision to submit the manuscript for publication.
