# Supplementary material for: Impact of adverse childhood experiences on analgesia-related outcomes: a systematic review
Source: Br J Anaesth. 2024 Oct 22;134(2):461–91. doi: 10.1016/j.bja.2024.09.015 (PMC11775844; doi:10.1016/j.bja.2024.09.015)
Supplement: Multimedia component 1 [file mmc1.docx]

# The impact of adverse childhood experiences on analgesia-related outcomes: a systematic review

DNS Senaratne, M Koponen, KN Barnett, BH Smith, TG Hales, L Marryat, LA Colvin

# Supplementary Material: Table of Contents

[Table S1: Search strategy 2](#_Toc165378752)

[Table S2: Study characteristics 5](#_Toc165378753)

[Table S3: Risk of bias assessment for observational studies (ROBINS-E) 11](#_Toc165378754)

[Table S4: Risk of bias assessment for interventional studies (RoB 2) 14](#_Toc165378755)

[Table S5: Study details on exposure – adverse childhood experiences 15](#_Toc165378756)

[Table S6: Commonly assessed adverse childhood experiences 37](#_Toc165378757)

Table S1: Search strategy

| # | APA PsycNET | CINAHL Plus via EBSCO | Cochrane CENTRAL | Embase via Ovid | MEDLINE via EBSCO | Scopus | Web of Science |
| --- | --- | --- | --- | --- | --- | --- | --- |
| 1 | (adverse childhood experiences) OR (advers* NEAR/2 child* NEAR/2 experienc*) | (adverse childhood experiences) OR (advers* N2 child* N2 experienc*) | (adverse childhood experiences) OR (advers* NEAR/2 child* NEAR/2 experienc*) | (adverse childhood experiences) OR (advers* adj2 child* adj2 experienc*) | (adverse childhood experiences) OR (advers* N2 child* N2 experienc*) | (adverse childhood experiences) OR (advers* w/1 child* w/1 experienc*) | (adverse childhood experiences) OR (advers* NEAR/2 child* NEAR/2 experienc*) |
| 2 | child* NEAR/2 (abus* OR advers* OR maltreat* OR neglect* OR trauma*) | child* N2 (abus* OR advers* OR maltreat* OR neglect* OR trauma*) | child* NEAR/2 (abus* OR advers* OR maltreat* OR neglect* OR trauma*) | child* adj2 (abus* OR advers* OR maltreat* OR neglect* OR trauma*) | child* N2 (abus* OR advers* OR maltreat* OR neglect* OR trauma*) | child* w/1 (abus* OR advers* OR maltreat* OR neglect* OR trauma*) | child* NEAR/2 (abus* OR advers* OR maltreat* OR neglect* OR trauma*) |
| 3 | #1 OR #2 | #1 OR #2 | #1 OR #2 | #1 OR #2 | #1 OR #2 | #1 OR #2 | #1 OR #2 |
| 4 | analges* OR painkill* OR (pain kill*) OR (pain medicat*) | analges* OR painkill* OR (pain kill*) OR (pain medicat*) | analges* OR painkill* OR (pain kill*) OR (pain medicat*) | analges* OR painkill* OR (pain kill*) OR (pain medicat*) | analges* OR painkill* OR (pain kill*) OR (pain medicat*) | analges* OR painkill* OR (pain kill*) OR (pain medicat*) | analges* OR painkill* OR (pain kill*) OR (pain medicat*) |
| 5 | (non-steroidal anti-inflammatory agent*) OR NSAID* OR opioid* OR opiate* OR gabapentinoid* OR (serotonin norepinephrine reuptake inhibitor*) OR SNRI* OR benzodiazepine* | (non-steroidal anti-inflammatory agent*) OR NSAID* OR opioid* OR opiate* OR gabapentinoid* OR (serotonin norepinephrine reuptake inhibitor*) OR SNRI* OR benzodiazepine* | (non-steroidal anti-inflammatory agent*) OR NSAID* OR opioid* OR opiate* OR gabapentinoid* OR (serotonin norepinephrine reuptake inhibitor*) OR SNRI* OR benzodiazepine* | (non-steroidal anti-inflammatory agent*) OR NSAID* OR opioid* OR opiate* OR gabapentinoid* OR (serotonin norepinephrine reuptake inhibitor*) OR SNRI* OR benzodiazepine* | (non-steroidal anti-inflammatory agent*) OR NSAID* OR opioid* OR opiate* OR gabapentinoid* OR (serotonin norepinephrine reuptake inhibitor*) OR SNRI* OR benzodiazepine* | (non-steroidal anti-inflammatory agent*) OR NSAID* OR opioid* OR opiate* OR gabapentinoid* OR (serotonin norepinephrine reuptake inhibitor*) OR SNRI* OR benzodiazepine* | (non-steroidal anti-inflammatory agent*) OR NSAID* OR opioid* OR opiate* OR gabapentinoid* OR (serotonin norepinephrine reuptake inhibitor*) OR SNRI* OR benzodiazepine* |
| 6 | carbamazepine OR gabapentin OR pregabalin OR oxcarbazepine OR lacosamide OR lamotrigine OR levetiracetam OR topiramate OR diazepam OR (valproic acid) OR duloxetine OR amitriptyline OR nortriptyline OR imipramine OR venlafaxine OR methoxyflurane OR paracetamol OR acetaminophen OR nefopam OR buprenorphine OR co-codamol OR codeine OR diamorphine OR dihydrocodeine OR dipipanone OR fentanyl OR hydromorphone OR meptazinol OR morphine OR oxycodone OR pentazocine OR pethidine OR tapentadol OR tramadol OR capsaicin OR co-dydramol OR methadone OR cannabidiol OR aceclofenac OR celecoxib OR dexketoprofen OR diclofenac OR etodolac OR etoricoxib OR felbinac OR flurbiprofen OR ibuprofen OR indometacin OR ketoprofen OR (mefenamic acid) OR meloxicam OR nabumetone OR naproxen OR piroxicam OR sulindac OR tenoxicam OR (tiaprofenic acid) OR (tolfenamic acid) OR ketamine | carbamazepine OR gabapentin OR pregabalin OR oxcarbazepine OR lacosamide OR lamotrigine OR levetiracetam OR topiramate OR diazepam OR (valproic acid) OR duloxetine OR amitriptyline OR nortriptyline OR imipramine OR venlafaxine OR methoxyflurane OR paracetamol OR acetaminophen OR nefopam OR buprenorphine OR co-codamol OR codeine OR diamorphine OR dihydrocodeine OR dipipanone OR fentanyl OR hydromorphone OR meptazinol OR morphine OR oxycodone OR pentazocine OR pethidine OR tapentadol OR tramadol OR capsaicin OR co-dydramol OR methadone OR cannabidiol OR aceclofenac OR celecoxib OR dexketoprofen OR diclofenac OR etodolac OR etoricoxib OR felbinac OR flurbiprofen OR ibuprofen OR indometacin OR ketoprofen OR (mefenamic acid) OR meloxicam OR nabumetone OR naproxen OR piroxicam OR sulindac or tenoxicam OR (tiaprofenic acid) OR (tolfenamic acid) OR ketamine | carbamazepine OR gabapentin OR pregabalin OR oxcarbazepine OR lacosamide OR lamotrigine OR levetiracetam OR topiramate OR diazepam OR (valproic acid) OR duloxetine OR amitriptyline OR nortriptyline OR imipramine OR venlafaxine OR methoxyflurane OR paracetamol OR acetaminophen OR nefopam OR buprenorphine OR co-codamol OR codeine OR diamorphine OR dihydrocodeine OR dipipanone OR fentanyl OR hydromorphone OR meptazinol OR morphine OR oxycodone OR pentazocine OR pethidine OR tapentadol OR tramadol OR capsaicin OR co-dydramol OR methadone OR cannabidiol OR aceclofenac OR celecoxib OR dexketoprofen OR diclofenac OR etodolac OR etoricoxib OR felbinac OR flurbiprofen OR ibuprofen OR indometacin OR ketoprofen OR (mefenamic acid) OR meloxicam OR nabumetone OR naproxen OR piroxicam OR sulindac or tenoxicam OR (tiaprofenic acid) OR (tolfenamic acid) OR ketamine | carbamazepine OR gabapentin OR pregabalin OR oxcarbazepine OR lacosamide OR lamotrigine OR levetiracetam OR topiramate OR diazepam OR (valproic acid) OR duloxetine OR amitriptyline OR nortriptyline OR imipramine OR venlafaxine OR methoxyflurane OR paracetamol OR acetaminophen OR nefopam OR buprenorphine OR co-codamol OR codeine OR diamorphine OR dihydrocodeine OR dipipanone OR fentanyl OR hydromorphone OR meptazinol OR morphine OR oxycodone OR pentazocine OR pethidine OR tapentadol OR tramadol OR capsaicin OR co-dydramol OR methadone OR cannabidiol OR aceclofenac OR celecoxib OR dexketoprofen OR diclofenac OR etodolac OR etoricoxib OR felbinac OR flurbiprofen OR ibuprofen OR indometacin OR ketoprofen OR (mefenamic acid) OR meloxicam OR nabumetone OR naproxen OR piroxicam OR sulindac or tenoxicam OR (tiaprofenic acid) OR (tolfenamic acid) OR ketamine | carbamazepine OR gabapentin OR pregabalin OR oxcarbazepine OR lacosamide OR lamotrigine OR levetiracetam OR topiramate OR diazepam OR (valproic acid) OR duloxetine OR amitriptyline OR nortriptyline OR imipramine OR venlafaxine OR methoxyflurane OR paracetamol OR acetaminophen OR nefopam OR buprenorphine OR co-codamol OR codeine OR diamorphine OR dihydrocodeine OR dipipanone OR fentanyl OR hydromorphone OR meptazinol OR morphine OR oxycodone OR pentazocine OR pethidine OR tapentadol OR tramadol OR capsaicin OR co-dydramol OR methadone OR cannabidiol OR aceclofenac OR celecoxib OR dexketoprofen OR diclofenac OR etodolac OR etoricoxib OR felbinac OR flurbiprofen OR ibuprofen OR indometacin OR ketoprofen OR (mefenamic acid) OR meloxicam OR nabumetone OR naproxen OR piroxicam OR sulindac or tenoxicam OR (tiaprofenic acid) OR (tolfenamic acid) OR ketamine | carbamazepine OR gabapentin OR pregabalin OR oxcarbazepine OR lacosamide OR lamotrigine OR levetiracetam OR topiramate OR diazepam OR (valproic acid) OR duloxetine OR amitriptyline OR nortriptyline OR imipramine OR venlafaxine OR methoxyflurane OR paracetamol OR acetaminophen OR nefopam OR buprenorphine OR co-codamol OR codeine OR diamorphine OR dihydrocodeine OR dipipanone OR fentanyl OR hydromorphone OR meptazinol OR morphine OR oxycodone OR pentazocine OR pethidine OR tapentadol OR tramadol OR capsaicin OR co-dydramol OR methadone OR cannabidiol OR aceclofenac OR celecoxib OR dexketoprofen OR diclofenac OR etodolac OR etoricoxib OR felbinac OR flurbiprofen OR ibuprofen OR indometacin OR ketoprofen OR (mefenamic acid) OR meloxicam OR nabumetone OR naproxen OR piroxicam OR sulindac or tenoxicam OR (tiaprofenic acid) OR (tolfenamic acid) OR ketamine | carbamazepine OR gabapentin OR pregabalin OR oxcarbazepine OR lacosamide OR lamotrigine OR levetiracetam OR topiramate OR diazepam OR (valproic acid) OR duloxetine OR amitriptyline OR nortriptyline OR imipramine OR venlafaxine OR methoxyflurane OR paracetamol OR acetaminophen OR nefopam OR buprenorphine OR co-codamol OR codeine OR diamorphine OR dihydrocodeine OR dipipanone OR fentanyl OR hydromorphone OR meptazinol OR morphine OR oxycodone OR pentazocine OR pethidine OR tapentadol OR tramadol OR capsaicin OR co-dydramol OR methadone OR cannabidiol OR aceclofenac OR celecoxib OR dexketoprofen OR diclofenac OR etodolac OR etoricoxib OR felbinac OR flurbiprofen OR ibuprofen OR indometacin OR ketoprofen OR (mefenamic acid) OR meloxicam OR nabumetone OR naproxen OR piroxicam OR sulindac or tenoxicam OR (tiaprofenic acid) OR (tolfenamic acid) OR ketamine |
| 7 | #4 OR #5 OR #6 | #4 OR #5 OR #6 | #4 OR #5 OR #6 | #4 OR #5 OR #6 | #4 OR #5 OR #6 | #4 OR #5 OR #6 | #4 OR #5 OR #6 |
| 8 | #3 AND #7 | #3 AND #7 | #3 AND #7 | #3 AND #7 | #3 AND #7 | #3 AND #7 | #3 AND #7 |

Table S2: Study characteristics

| **First Author & Year** | **Study Design** | **Participant Population** | **Population Country** | **Years of Data Collection** | **Sample Size** | **Female Gender (%)** | **Age** |
| --- | --- | --- | --- | --- | --- | --- | --- |
| Afifi 2012 | Cross-sectional | National Epidemiological Study of Alcohol and Related Conditions (NESARC), wave 2: a nationwide nationally representative household survey of adults. | USA | 2001-02; 2004-05 | 34653 | NR | NR |
| Alexander 1998 | Cross-sectional | Women with fibromyalgia attending an outpatient rheumatology clinic. | USA | 1992-94 | 75 | 100.0% | Mean 46.4*; SD 9.8* |
| Ararso 2021 | Cohort | National Longitudinal Study of Adolescent to Adult Health (Add Health), waves 1, 3, and 4: a nationally representative cohort of adolescents aged 12-19 recruited from schools and followed up for over 20 years. | USA | 1994-95; 2001-02; 2007-08 | 12288 | 52.0% | Wave 1: Range 11-21 Wave 3: Range 18-26 Wave 4: Range 24-32 |
| Austin 2018a | Cohort | National Longitudinal Study of Adolescent to Adult Health (Add Health), waves 1 and 4: a nationally representative cohort of adolescents aged 12-19 recruited from schools and followed up for over 20 years. | USA | 1994-95; 2007-08 | 14800 | 53.2% | Range 24-32 |
| Austin 2018b | Cohort | National Longitudinal Study of Adolescent to Adult Health (Add Health), waves 1 and 3: a nationally representative cohort of adolescents aged 12-19 recruited from schools and followed up for over 20 years. | USA | 1994-95; 2001-02 | 14322 | 52.9% | Wave 3: Range 18-26; mean 15.5*; SD 11.9* |
| Baumann-Larsen 2023 | Cross-sectional | Trøndelag Health Study (HUNT), Young-HUNT3 and HUNT4: adolescents aged 13-19 living in the Nord-Trøndelag region (Young-HUNT3) followed up as adults (HUNT4). | Norway | 2006-08; 2017-19 | 2947 | 59.1% | Young-HUNT3: mean 16.0; SD 1.8 HUNT4: mean 27.1; SD 1.9 |
| Bottiroli 2019 | Cross-sectional | Adults with chronic migraine and medication overuse headache attending an inpatient detoxification clinic. | Italy | 2014-16 | 166 | 79.5% | Mean 44.7*; SD 10.0* |
| Browne 1998 | Cross-sectional | Adults attending the outpatient National Drug Treatment Centre. | Republic of Ireland | 1997 | 52 | 46.2% | Range 16-60; mean 27; SD 8.9 |
| Carlyle 2021 | Double-blind, placebo-controlled, cross-over randomised controlled trial | Adults recruited through "...convenience and snowball sampling via participant databases, poster advertisements, and word of mouth…". | UK | NR | 52 | 67.3% | Range 18-65; mean 30.9; SD 14.9 |
| Carlyle 2023 | Cross-sectional | Healthy adults (American Society of Anaesthesiologists grade 1-2) attending a day-case surgery unit. | Norway | 2018-21; 2021-22 | 155 | 51.7% | Range 17-81; median 48.0; mean 47.9; SD 14.0 |
| Carr 2023 | Cross-sectional | Adults with opioid use disorder attending an outpatient addiction treatment centre. | USA | NR | 171 | 56.7% | Mean 51.5; SD 14.9 |
| Conroy 2009 | Case-control | Cases: Adults attending opioid pharmacotherapy clinics. Controls: Adults recruited through public advertising. | Australia | 2005-07 | Cases: 967 Controls: 346 | Cases: 39.0% Controls: 55.0% | Cases: Mean 36.5 Controls: Mean 34.7 |
| Davis 2022 | Cohort | Adolescents recruited from 16 middle schools and followed up annually for 12 years, waves 8-12. | USA | 2008; 2016-19 | 2880 | 54.9% | Wave 8: Range 17-20; mean 18.3; SD 0.8 |
| Derefinko 2019 | Cross-sectional | Adults attending an outpatient opioid use disorder clinic. | USA | 2011-17 | 87 | 25.3% | Range 21-81; mean 39.9; SD 9.5 |
| Dunn 2022 | Cross-sectional | Adults with a history of heroin or prescription opioid use recruited online through Amazon Mechanical Turk. | USA | 2020 | 310 | 58.4% | Mean 38.6; SD 11.9 |
| Eaves 2021 | Cross-sectional | Adults incarcerated at a county detention facility. | USA | 2017-18 | 96 | 26.0% | 18-24: 10/96 (10.4%) 25-34: 39 (40.6%) 35-44: 25 (26.0%) 45-54: 17 (17.7%) ≥55: 5 (5.2%) |
| El-Bassel 2019 | Cross-sectional | Women participating in Project PACT (a couple-focused randomized clinical trial of an HIV prevention intervention for men undergoing community corrections and their female intimate partners) who reported lifetime use of illicit drugs. | USA | NR | 201 | 100.0% | Median 34.8; IQR 23.1-64.9 |
| Elhammady 2014 | Case-control | Cases: Adults with opioid dependence syndrome, heroin dependence, or methadone prescription attending an outpatient addiction clinic. Controls: Friends and family members of cases. | UK | NR | Cases: 120 Controls: 100 | 25.0% | Cases: Range 20-65; mean 33.3; SD 8.8 Controls: Range 20-54; mean 36.5; SD 9.5 |
| Fortson 2021 | Cross-sectional | Adults attending a university. | USA | 2019 | 1402 | 74.8% | Mean 23.5; SD 6.5 |
| Fuss 2023 | Cohort | Vape shop Advertising, Place characteristics, and Effects Surveillance study (VAPES): adults aged 18-34 recruited online through Facebook and Reddit and followed up for 1 year. | USA | 2018-19 | 2975 | 55.0% | Range 18-34; mean 24.6; SD 4.7 |
| Garami 2019 | Case-control | Cases: Adults with a history of opiate addiction attending an opioid treatment program. Controls: Adults recruited via word of mouth from the community. | Australia | NR | Cases: 36 Controls: 33 | Cases: 75.0% Controls: 63.6% | Cases: Mean 44.7; SD 9.4 Controls: Mean 39.5; SD 14.9 |
| Garland 2019 | Cross-sectional | Women with chronic pain and regular opioid analgesic use in the last 90 days attending primary care and specialty pain clinics. | USA | NR | 36 | 100.0% | Mean 51.2; SD 9.5 |
| Griego 2022 | Cross-sectional | Women with chronic pelvic pain attending outpatient OB/GYN or pain clinics. | USA | 2015-20 | 113 | 100.0% | Mean 44; SD 14 |
| Groh 2020 | Cross-sectional | Adults with opioid dependency attending a diamorphine maintenance treatment clinic. | Germany | NR | 15 | 26.7% | Mean 44.8* |
| Guarino 2021 | Cross-sectional | Adults aged 18-29 with prescription opioid or heroin use in the preceding 30 days. | USA | 2014-16 | 539 | 31.5% | Range 18-29; mean 24.5; SD 3.1 |
| Heffernan 2000 | Cross-sectional | Adults attending an inpatient psychiatric hospital. | USA | 1991-92 | 763 | 52.9% | Range 18-59 |
| Hill 2022 | Cross-sectional | Adults recruited through public advertising. | USA | NR | 75 | 52.0% | Range 18-25; mean 21.1, SD 2.2 |
| Khoury 2010 | Cross-sectional | Adults attending outpatient general medical and OB/GYN clinics. | USA | NR | 587 | 61.2% | Mean 42.4; SD 12.7 |
| Kors 2022 | Cross-sectional | Pregnant women (2nd trimester or later) attending a high-risk pregnancy clinic. | USA | NR | 93 | 100.0% | Mean 27.2; SD 4.3 |
| Kumar 2016 | Cross-sectional | Adults attending an outpatient buprenorphine treatment program. | USA | 2012-15 | 113 | 44.0% | Mean 32.1; SD 9.3 |
| Larance 2018 | Cross-sectional | Adults with a history of heroin dependence receiving opioid agonist treatment. | Australia | 2004-08 | 1149 | 38.6% | Mean 36.3; SD 8.5 |
| Lee 2023 | Cross-sectional | Midlife in the United States study (MIDUS), wave 2: a nationally representative sample of adults. | USA | 2004-06 | 865 | 59.8% | Range 35-84; mean 59.6*; SD 11.5* |
| Lovallo 2018 | Cross-sectional | Oklahoma Family Health Patterns Project: healthy women aged 18-30. | USA | NR | 72 | 100.0% | Range 18-30; mean 23.2*; SD 2.8* |
| Lynskey 2006 | Cross-sectional | The Australian Twin Study: Twins recruited from schools in Australia and followed up through adulthood. | Australia | 1980-82; 1996-2000 | 6265 | NR | Range 24-36; median 30 |
| Martin 2023 | Cross-sectional | Adults with substance use disorder recruited via media advertising. | USA | NR | 565 | 49.9% | Mean 33.4; SD 10.1 |
| McDonagh 2023 | Cross-sectional | Adults attending substance use services. | Republic of Ireland | 2017; 2019 | 104 | 36.5% | Mean 42.7; SD 7.4 |
| Meadows 2023 | Cross-sectional | Adults recruited through public advertising. | USA | 2018 | 107 | 33.6% | Mean 36.6; SD 11.4 |
| Merrick 2020 | Cross-sectional | Behavioural Risk Factor Surveillance System (BRFSS), Montana and Florida populations: adults responding to a telephone survey. | USA | Montana: 2011 Florida: 2010 | Montana: 8726 Florida: 27545 | Montana: 50.3% Florida: 51.4% | Montana: 18-24: 436/8726 (8.4% weighted) 25-34: 794/8726 (6.2% weighted) 35-44: 932/8726 (4.4% weighted) 45-54: 1584/8726 (4.1% weighted) 55-64: 2173/8726 (1.5% weighted) ≥64: 2722/8726 (0.7% weighted) Florida: 18-24: 609/27545 (7.5% weighted) 25-34: 1698/27545 (3.1% weighted) 35-44: 2726/27545 (2.5% weighted) 45-54: 4644/27545 (1.9% weighted) 55-64: 6559/27545 (1.3% weighted) ≥64: 11426/27545 (1.7% weighted) |
| Mirhashem 2017 | Cross-sectional | Adults with a history of opioid use >1 year. | USA | NR | 84 | 46.4% | Mean 35.3, SD 8.3 |
| Myers 2014 | Cross-sectional | National Epidemiological Study of Alcohol and Related Conditions (NESARC), wave 2: a nationwide nationally-representative household survey of adults. | USA | 2001-02; 2004-05 | 34653 | NR | 20-29: 4913 (16.3% weighted) 30-39: 6621 (18.7% weighted) 40-49: 7539 (21.5% weighted) 50-59: 6117 (17.7% weighted) 60-69: 4174 (11.5% weighted) ≥70: 5289 (14.2% weighted) |
| Naqavi 2011 | Case-control | Cases: Opiate dependent adults attending drug treatment centres. Controls: Adults who visited neighbourhood clinics for reasons other than addiction treatment. | Iran | NR | Cases: 212 Controls: 216 | Cases: 15.6% Controls 23.1% | Cases: Mean 31.4; SD 6.7 Controls: Mean 30.8; SD 7.5 |
| Nelson 2006 | Cross-sectional | The Australian Twin Study: Twins recruited from schools in Australia and followed up through adulthood. | Australia | 1996-2000 | 6050 | 55.5% | Mean 29.9; SD 2.5 |
| Onu 2021 | Cross-sectional | Adults attending a university, recruited from local student accommodation and hostels. | Nigeria | NR | 301 | 4.0% | Mean 22.6; SD 3.5 |
| Pakdaman 2021 | Cross-sectional | Adults attending a university. | USA | 2018 | 3899 | 69.9% | Mean 24.6; SD 7.7 |
| Pierce 2019 | Cross-sectional | Adults with current opioid use attending an outpatient pain clinic. | USA | 2011-16 | 1785 | 57.8% | Mean 50.3; SD 14.8 |
| Pierce 2020 | Cross-sectional | Adults attending an outpatient pain clinic. | USA | 2011-16 | 3118 | 59.5% | Mean 50.3; SD 15.3 |
| Quinn 2016 | Cohort | National Longitudinal Study of Adolescent to Adult Health (Add Health), waves 3, and 4: a nationally representative cohort of adolescents aged 12-19 recruited from schools and followed up for over 20 years. | USA | 1994-95; 2001-02; 2007-08 | 12288 | 54.4% | Wave 3: Range 18-26  Wave 4: Range 24-34 |
| Roy 2002 | Cross-sectional | Adults with opiate dependency attending substance abuse clinics. | USA | NR | 246 | 12.6% | Mean 44.3*; SD 7.8* |
| Sansone 2010 | Cross-sectional | Adults attending an outpatient internal medicine clinic. | USA | NR | 80 | 73.8% | Range 17-74; mean 45.6; SD 14.7 |
| Santo Jr 2022 | Cross-sectional | Pain and Opioids in Treatment (POINT) study: adults prescribed regulated opioids for chronic non-cancer pain recruited from community pharmacies. | Australia | 2012 | 1514 | 55.6% | Mean 57.4; SD 13.7 |
| Sartor 2014 | Cross-sectional | Adults with opioid dependence attending for an alternative research study. | USA | NR | 3513 | 38.0% | Mean 39.1; SD 10.0 |
| Smith 2022 | Cross-sectional | Adults with a history of alcohol, prescription opioid, illicit opioid, kratom, or illicit stimulant use in the last 6 months, recruited through Amazon Mechanical Turk. | USA | 2020 | 1510 | 59.9% | Mean 36.4; SD 10.7 |
| Stein 2017 | Cross-sectional | Adults attending an inpatient opioid detoxification program. | USA | 2015 | 457 | 28.7% | Range 18-64; median 30; mean 32.2; SD 8.6 |
| Tang 2020 | Cross-sectional | National Epidemiological Study of Alcohol and Related Conditions (NESARC), wave 3: a nationwide nationally representative household survey of adults. | USA | 2012-13 | 36309 | 56.3% | Mean 45.6; SD 17.5 |
| Taplin 2014 | Cross-sectional | Adults with a history of opioid injection who had previously participated in the North American Opiate Medication Initiative (NAOMI). | USA | 2008 | 87 | 41.4% | Mean 38.0 |
| Testa 2023 | Cross-sectional | Pregnancy Risk Assessment Monitoring System (PRAMS), North Dakota and South Dakota populations: routine surveillance system for mothers who have recently given birth. | USA | 2019-20 | 2999 | 100.0% | ≤24: 601*/2999 (20.0%) 25-29: 1030*/2999 (34.3%) 30-34: 951*/2999 (31.7%) ≥35: 417*/2999 (13.9%) |
| Thiesset 2023 | Cross-sectional | Adults with a history of opioid use disorder identified through the University of Utah's health system's electronic data warehouse. | USA | NR | 124 | 51.6% | 18-29: 13*/124 (10.4%*) 30-39: 23*/124 (18.5%*) 40-49: 26*/124 (21.0%*) 50-59: 19*/124 (15.3%*) 60-69: 21*/124 (16.9%*) ≥70: 11*/124 (8.9%*) |
| Tomassi 2017 | Cross-sectional | Adults aged 18-54 with first presentation of psychosis attending community mental health centres. | Italy | 2010-11 | 345 | 42.3% | Mean 29.8; SD 9.7 |
| Vogel 2011 | Cross-sectional | Adults with opioid dependence attending outpatient clinics. | Switzerland | NR | 193 | 33.7% | Mean 42; SD 7 |
| Wang 2021 | Cross-sectional | National Epidemiological Study of Alcohol and Related Conditions (NESARC), wave 3: a nationwide nationally representative household survey of adults. | USA | 2012-13 | 33613 | 52.0% | Mean 45.7; SD 17.8 |
| Widom 2006 | Cohort | Children <12 years with court-substantiated abuse/neglect between 1967-71 and matched non-abused controls, followed up for over 20 years. | USA | 1967-71; 1989-95; 2000-02 | 892 | 51.0% | Range 30-47; mean 39.5; SD 3.5 |
| Williams 2020 | Cross-sectional | Adults with a history of at least one type of inter-personal trauma (intimate partner violence, sexual assault, and/or ACEs) recruited through public advertising. | USA | 2018 | 230 | 61.7% | Mean 28.6; SD 10.9 |
| Williams 2021 | Cross-sectional | Adults with a history of at least one type of inter-personal trauma (intimate partner violence, sexual assault, and/or ACEs) recruited through public advertising. | USA | 2018 | 235 | 62.6% | Mean 29.0; SD 11.0 |
| Wuest 2007 | Cross-sectional | Women's Health Effects Study (WHES): English speaking women who had left an abusive partner in the preceding 3-36 months and who had a positive Abuse Assessment Screen. | Canada | NR | 309 | 100.0% | Range 19-63; mean 39.4; SD 9.8 |
| You 2019 | Cross-sectional | Adults attending a university. | USA | NR | 3073 | 72.3% | Mean 18.8; SD 1.4 |
| Zehetmeier 2023 | Cohort | Pregnant women (3rd trimester) attending an inpatient obstetric clinic. | Germany | 2020-21 | 191 | 100.0% | Range 20-46; mean 32.3*; SD 4.3* |

NR = not reported, SD = standard deviation. *Value calculated from data presented in paper.

Table S3: Risk of bias assessment for observational studies (ROBINS-E)

| **Author & Year** | **Domain 1** | **Domain 2** | **Domain 3** | **Domain 4** | **Domain 5** | **Domain 6** | **Domain 7** | **Overall** |
| --- | --- | --- | --- | --- | --- | --- | --- | --- |
| Afifi 2012 | High | Some Concerns | Some Concerns | Low | High | Low | Low | High |
| Alexander 1998 | High | High | Very High | Low | Low | Low | Low | Very High |
| Ararso 2021 | High | Some Concerns | Some Concerns | Low | High | Low | Low | High |
| Austin 2018a | Low | High | Some Concerns | Low | Low | Low | Low | High |
| Austin 2018b | Low | Some Concerns | Some Concerns | Low | Low | Low | Low | Some Concerns |
| Baumann-Larsen 2023 | Very High | Some Concerns | Some Concerns | Low | Some Concerns | Low | Low | Very High |
| Bottiroli 2019 | High | Some Concerns | Very High | Low | High | Low | Low | Very High |
| Browne 1998 | High | High | Very High | Low | High | Low | Low | Very High |
| Carlyle 2023 | High | Some Concerns | Some Concerns | Low | Low | Low | Low | High |
| Carr 2023 | High | Low | Very High | Low | High | Some Concerns | High | Very High |
| Conroy 2009 | High | Some Concerns | Very High | Low | High | Some Concerns | High | Very High |
| Davis 2022 | High | Some Concerns | Some Concerns | Low | Low | Low | Low | High |
| Derefinko 2019 | Some Concerns | Low | Very High | Low | Low | Low | Low | Very High |
| Dunn 2022 | High | Some Concerns | Very High | Low | High | Low | Low | Very High |
| Eaves 2021 | Some Concerns | Some Concerns | Very High | Low | High | Low | Low | Very High |
| El-Bassel 2019 | High | Some Concerns | Very High | Low | High | Low | Low | Very High |
| Elhammady 2014 | High | Some Concerns | Very High | Low | High | Low | Low | Very High |
| Fortson 2021 | High | Low | High | Low | High | Some Concerns | Low | Very High |
| Fuss 2023 | High | Low | Very High | Low | High | Low | Low | Very High |
| Garami 2019 | High | Some Concerns | Very High | Low | High | Low | Low | Very High |
| Garland 2019 | High | Low | Very High | Low | High | Low | Low | Very High |
| Griego 2022 | High | Some Concerns | Very High | Low | Very High | Low | Low | Very High |
| Groh 2020 | High | Some Concerns | Some Concerns | Low | High | Low | Low | High |
| Guarino 2021 | High | Low | High | Low | Low | Some Concerns | Low | High |
| Heffernan 2000 | High | High | Very High | Low | High | Low | Low | Very High |
| Hill 2022 | High | Some Concerns | Some Concerns | Low | High | Low | Low | High |
| Khoury 2010 | High | High | Very High | Low | High | Low | Low | Very High |
| Kors 2022 | High | Some Concerns | Very High | Low | High | Low | Low | Very High |
| Kumar 2016 | High | Some Concerns | Very High | Low | Some Concerns | Low | Low | Very High |
| Larance 2018 | High | Some Concerns | Very High | Low | High | Low | Low | Very High |
| Lee 2023 | High | High | High | Low | Low | Low | Low | High |
| Lovallo 2018 | High | High | Some Concerns | Low | Low | Low | Low | High |
| Lynskey 2006 | High | High | Some Concerns | Low | High | Low | Low | Very High |
| Martin 2023 | High | Low | Very High | Low | Low | Low | Low | Very High |
| McDonagh 2023 | High | Low | Very High | Low | Low | Low | Low | Very High |
| Meadows 2023 | High | Low | Very High | Low | Low | Low | Low | Very High |
| Merrick 2020 | High | Some Concerns | Some Concerns | Low | High | Some Concerns | Low | High |
| Mirhashem 2017 | High | Some Concerns | Very High | Low | High | Low | Low | Very High |
| Myers 2014 | High | Low | Some Concerns | Low | High | Low | Low | High |
| Naqavi 2011 | High | Some Concerns | Very High | Low | High | Low | Low | Very High |
| Nelson 2006 | High | High | Some Concerns | Low | High | Low | Low | Very High |
| Onu 2021 | High | Low | Very High | Low | High | Low | Low | Very High |
| Pakdaman 2021 | Some Concerns | Some Concerns | Some Concerns | Low | Some Concerns | Low | Low | High |
| Pierce 2019 | High | High | Very High | Low | Low | Low | Low | Very High |
| Pierce 2020 | High | High | Very High | Low | Low | Low | Low | Very High |
| Quinn 2016 | High | Low | Some Concerns | Low | High | Some Concerns | Low | High |
| Roy 2002 | High | Some Concerns | Very High | Low | High | Low | Some Concerns | Very High |
| Sandoe 2022 | High | Low | Very High | Low | High | Low | Some Concerns | Very High |
| Sansone 2010 | High | Some Concerns | Very High | Low | High | Low | Low | Very High |
| Santo Jr 2022 | High | Low | Some Concerns | Low | Low | Low | Low | High |
| Sartor 2014 | High | High | Very High | Low | High | Low | Low | Very High |
| Smith 2021 | High | Low | Very High | Low | Low | Low | Low | Very High |
| Stein 2017 | Some Concerns | Low | Very High | Low | High | Low | Low | Very High |
| Tang 2020 | High | Low | Some Concerns | Low | High | Low | Low | High |
| Taplin 2014 | High | Some Concerns | Very High | Low | High | Low | Some Concerns | Very High |
| Testa 2023 | High | Low | Some Concerns | Low | High | Low | Low | High |
| Thiesset 2023 | High | Low | Very High | Low | Low | Low | Low | Very High |
| Tomassi 2017 | High | High | Very High | Low | High | Low | Low | Very High |
| Vogel 2011 | High | Some Concerns | Very High | Low | High | Low | Some Concerns | Very High |
| Wang 2021 | High | Low | Some Concerns | Low | Low | Low | Low | High |
| Widom 2006 | Low | High | Some Concerns | Low | High | Low | Low | High |
| Williams 2020 | High | Some Concerns | Very High | Low | Low | Low | Low | Very High |
| Williams 2021 | High | Some Concerns | Very High | Low | High | Low | Low | Very High |
| Wuest 2007 | High | High | Very High | Low | High | Low | Low | Very High |
| You 2019 | High | Low | Some Concerns | Low | Low | Low | Low | High |
| Zehetmeier 2023 | High | Some Concerns | Some Concerns | Low | High | Low | Low | High |

Domain 1 = risk of bias due to confounding, domain 2 = risk of bias arising from measurement of the exposure, domain 3 = risk of bias in selection of participants into the study, domain 4 = risk of bias due to post-exposure interventions, domain 5 = risk of bias due to missing data, domain 6 = risk of bias arising from measurement of outcomes, domain 7 = risk of bias in selection of the reported result. Overall risk of bias rating was determined by the ROBINS-E algorithm. This was usually the highest rating across all domains, however if ≥3 domains shared the highest rating then the overall assessment was upgraded to a higher risk level (e.g. ≥3 “High” ratings in individual domains led to a “Very High” overall rating).

Table S4: Risk of bias assessment for interventional studies (RoB 2)

| **Author & Year** | **Domain 1** | **Domain 2** | **Domain 3** | **Domain 4** | **Domain 5** | **Overall** |
| --- | --- | --- | --- | --- | --- | --- |
| Carlyle 2021 | Some Concerns | Low | Low | Low | Some Concerns | Some Concerns |

Domain 1 = risk of bias arising from the randomisation process, domain 2 = risk of bias due to deviations from the intended interventions, domain 3 = risk of bias due to missing outcome data, domain 4 = risk of bias in measurement of the outcome, domain 5 = risk of bias in selection of the reported result. Overall risk of bias rating was determined by the RoB 2 algorithm. This was usually the highest rating across all domains.

Table S5: Study details on exposure – adverse childhood experiences

| **Author & Year** | **Exposure** | **Exposure Definition** | **Exposure Tool** | **Upper Age Limit of Childhood** | **Number of Adversities** | **List of Adversities** | **Adversity Prevalence** |
| --- | --- | --- | --- | --- | --- | --- | --- |
| Afifi 2012 | Childhood maltreatment | NR | Structured interview using questions from Conflict Tactics Scale and the Childhood Trauma Questionnaire. | < 18 | 5 | emotional abuse; physical abuse; sexual abuse; physical neglect; emotional neglect | NR |
| Alexander 1998 | Sexual abuse & physical abuse | NR | Structured interview using "… 7 questions regarding subjects' experiences with sexual/physical abuse; the questions we used were identical to those used by Drossman and colleagues…". | < 14 | 2 | sexual abuse; physical abuse | 0: 32/75 (42.7%) ≥1: 43/75 (57.3%)  Sexual abuse: 40*/75 (53.3%*) Physical abuse: 16*/75 (21.3%*) |
| Ararso 2021 | Child abuse and homelessness | "…child abuse included both physical and/or sexual abuse..." | Structured interview using Add Health cohort questions. | < 18 | 2 | physical and/or sexual abuse; homelessness | Physical and/or sexual abuse: 2202/11607* (18.5%) Homelessness: 475/12275* (3.9%) |
| Austin 2018a | Childhood abuse | NR | Structured interview using Add Health cohort questions. | < 18 | 3 | emotional abuse; physical abuse; sexual abuse | 0: 7281*/14714* (49.5%*) ≥1: 7433*/7281* (50.5%*)  Emotional abuse: 6921*/14587* (47.4%*) Physical abuse: 2712*/14627* (18.5%*) Sexual abuse: 751*/14651* (5.1%*) |
| Austin 2018b | Childhood abuse & neglect | NR | Structured interview using Add Health cohort questions, with ACE questions being combined into a single childhood abuse & neglect latent variable. | < 13 | 4 | physical abuse; sexual abuse; supervisory neglect; physical neglect | Physical abuse: 3971*/14083* (28.2%*) Sexual abuse: 630*/14083* (4.5%*) Supervisory neglect: 5337*/14083* (37.9%*) Physical neglect: 1537*/14083* (10.9%*) |
| Baumann-Larsen 2023 | Potentially traumatic events | NR | Self-completed questionnaire using "...a brief lifetime trauma screen derived from the UCLA PTSD Index for DSM IV, part I…adapted to a Norwegian context...". | NR | 6 | physical violence; bullying; sexual abuse; witness to violence; severe illness or death of someone close; severe accident, disaster, or other traumatic event | Bullying: 202*/2911* (6.9%*) Physical violence: 239*/2911* (8.2%*) Sexual abuse: 147*/2911* (5.0%*) Witness to violence: 578*/2911* (19.9%*) Severe illness or death of someone close: 2052*/2911* (70.5%) Severe accident, disaster, or other traumatic event: 847*/2911* (29.1%*) |
| Bottiroli 2019 | Childhood trauma | "...such as physical and emotional abuse and neglect…" | Self-completed questionnaire using a shortened Italian version of the Childhood Trauma Questionnaire. | NR | 5 | emotional abuse; emotional neglect; physical abuse; physical neglect; sexual abuse | Total number of events: mean 1.2*; SD 1.3*  Emotional trauma events: mean 0.8; SD 1.0 Physical trauma events: mean 0.4; SD 0.7  Sexual abuse (yes/no): 11*/166 (6.6%*) |
| Browne 1998 | Sexual abuse & physical abuse | NR | Semi-structured interview involving "…a review of early life experiences…". | NR | 2 | sexual abuse; physical abuse | Sexual abuse: 11/52 (21.2%*) Physical abuse: 12/52 (23.1%*) |
| Carlyle 2021 | Childhood trauma | NR | Self-completed online questionnaire using the Childhood Trauma Questionnaire | NR | 5 | emotional abuse; physical abuse; sexual abuse; emotional neglect; physical neglect | High trauma: Total score: mean 64.4; SD 13.6 Physical abuse score: mean 11.6; 5.5 Emotional abuse score: mean 16.4; SD 4.8 Sexual abuse score: mean 9.2; SD 5.9 Physical neglect score: mean 9.9; SD 3.8 Emotional neglect score: mean 16.7; SD 4.1  No trauma: Total score: mean 28.2; SD 2.6 Physical abuse score: mean 5.1; 0.3 Emotional abuse score: mean 6.0; SD 1.0 Sexual abuse score: mean 5.1; SD 0.4 Physical neglect score: mean 5.3; SD 0.6 Emotional neglect score: mean 6.7; SD 1.6 |
| Carlyle 2023 | Childhood adversity | "…such as abuse, neglect, and household dysfunction…" | Self-completed questionnaire using the Childhood Trauma Questionnaire. | NR | 5 | emotional abuse; physical abuse; emotional neglect; physical neglect; sexual abuse | Mean 33.1; SD 9.8  Physical abuse: mean 5.4; SD 1.3 Physical neglect: mean 6.3; SD 2.1 Emotional abuse: mean 7.1; SD 3.4 Emotional neglect: mean 8.7; SD 3.8 Sexual abuse: 5.7; SD 2.7 |
| Carr 2023 | Childhood adversity | NR | Self-completed questionnaire using the Adverse Childhood Experiences Questionnaire. | < 18 | 10 | verbal abuse; physical abuse; sexual abuse; emotional abuse; physical neglect; parental separation/divorce; domestic violence; household substance misuse; household mental illness; household member incarceration  Source of the list of ACEs was cited as Felitti et al. 1998, who measured 7 ACEs using 17 questions, and did not measure verbal abuse (as distinct from emotional/psychological abuse), physical neglect, or parental separation/divorce. | Mean 3.4*; SD 2.7*  Verbal abuse: 69*/171 (40.4%*) Physical abuse: 58*/171 (33.9%*) Sexual abuse: 44*/171 (25.7%*) Emotional abuse: 52*/171 (30.4%*) Physical neglect: 29*/171 (17.0%*) Parental separation/divorce: 105*/171 (61.4%*) Domestic violence: 51*/171 (29.8%*) Household substance misuse: 86*/171 (50.3%*) Household mental illness: 43*/171 (25.1%*) Household member incarceration: 45*/171 (26.3%*) |
| Conroy 2009 | Childhood maltreatment | "...in terms of sexual abuse, physical abuse, emotional abuse and neglect..." | Structured interview using questions from: 1) the Christchurch Health and Development Study; and 2) Strauss' conceptualization of neglect. | < 18 | 4 | sexual abuse; physical abuse; neglect; emotional abuse | Sexual abuse: 645*/1313* (49.1%*) Physical abuse: 699*/1313* (53.2%*) Emotional abuse: 644*/1313* (59.9%*) Neglect: 920*/1313* (70.1%*) |
| Davis 2022 | Victimization | NR | Self-completed online questionnaire using items derived from the Adverse Childhood Experiences Questionnaire, with subsequent latent class analysis identifying 4 classes. | < 18 | 4 | emotional abuse; physical abuse; sexual abuse; domestic violence | NR |
| Derefinko 2019 | Adverse childhood experiences | "...emotional/physical/sexual abuse, neglect, use of drugs in the household, divorce, familial mental illness, and imprisoned family members before the age of 18…" | Initial clinical assessment using the Adverse Childhood Experiences Questionnaire. | < 18 | 10 | emotional abuse; physical abuse; sexual abuse; neglect; family dysfunction  Full list not reported. Source of the list of ACEs was cited as Anda et al. 1999, who measured 8 ACEs using 18 questions. | 0: 16/87 (18.4%) 1-3: 31/87 (35.6%) ≥4: 40/87 (46.0%) |
| Dunn 2022 | Early life trauma | "…general, physical, emotional, and/or sexual events that occur before the age of 18, which may alter normal development and have the potential to confer long-lasting harmful effects on the individual's physical and psychological functioning…" | Self-completed online questionnaire using the Early Life Trauma Inventory Self Report questionnaire. | < 18 | 4 | general trauma; physical trauma; emotional trauma; sexual trauma | Total trauma score: range 0 to 27; mean 9.9; SD 5.9  0: 22*/310 (7.1%) ≥1: 288*/310 (92.9%)  General trauma score: range 0 to 11; mean 3.7; SD 2.5 Physical trauma score: range 0 to 5; mean 2.3; SD 1.5 Emotional trauma score: range 0 to 5; mean 2.2; SD 1.7 Sexual trauma score: range 0 to 6; mean 1.6; SD 1.8 |
| Eaves 2021 | Adverse childhood experiences | NR | Self-completed questionnaire using the Behavioral Risk Factor Surveillance System ACE module. | < 18 | 9 | physical abuse; emotional abuse; sexual abuse; parental separation/divorce; witnessed domestic violence; household member mental illness/suicide; household alcohol misuse; household substance misuse; household member incarceration | Household member mental illness: 30*/96 (31.3%*) Household alcohol misuse: 49*/96 (51.0%*) Household substance misuse: 34*/96 (35.4%*) Household member incarceration: 40*/96 (41.7%*) Parental separation/divorce: 53*/96 (55.2%*) Witnessed domestic violence: 52*/96 (54.2%*) Physical abuse: 47*/96 (49.0%*) Emotional abuse: 66*/96 (68.8%*) Sexual abuse: 28*/96 (29.2%*) |
| El-Bassel 2019 | Childhood adversity | NR | Structured interview using the Revised Inventory of Adverse Childhood Experiences. | < 17 | 6 | emotional abuse; sexual abuse; physical abuse; domestic violence; foster care; running away from home | Total ACE count: median 2.3; IQR 0-6 |
| Elhammady 2014 | Child sexual abuse, child physical abuse, and parental history of drug/alcohol misuse | NR | Semi-structured interview using the Life Events Checklist. | < 16 | 3 | sexual abuse; physical abuse; parental alcohol/substance misuse | Cases: Sexual abuse: 21/120 (17.5%) Physical abuse: 32/120 (26.7%) Parental drug/alcohol misuse: 26/120 (21.7%)  Controls: Sexual abuse: 11/100 (11.0%) Physical abuse: 15/100 (15.0%) Parental drug/alcohol misuse: 11/100 (11.0%) |
| Fortson 2021 | Adverse childhood experiences | NR | Self-completed online questionnaire using the Adverse Childhood Experiences Questionnaire. | NR | 10 | emotional abuse; physical abuse; sexual abuse; emotional neglect; physical neglect; parental separation/divorce; domestic violence; household alcohol/substance misuse; household mental illness; household member incarceration | 0: 547*/1402 (39.0%) 1-3: 610*/1402 (43.5%) ≥4: 245*/1402 (17.5%)  Emotional abuse: 314/1402 (22.4%) Physical abuse: 161/1402 (11.5%) Sexual abuse: 134/1402 (9.6%) Emotional neglect: 267/1402 (19.0%) Physical neglect: 62/1402 (4.4%) Parental separation/divorce: 428/1402 (30.5%) Domestic violence: 96/1402 (6.8%) Household alcohol/substance misuse: 286/1402 (20.4%) Household mental illness: 456/1402 (32.5%) Household member incarceration: 76/1402 (5.4%) |
| Fuss 2023 | Adverse childhood experiences | NR | Self-completed online questionnaire using the Adverse Childhood Experiences Questionnaire. | < 18 | 10 | List not reported. Source of the list of ACEs was cited as Felitti et al. 1998, who measured 7 ACEs using 17 questions. | Mean 2.1; SD 2.3 |
| Garami 2019 | Childhood trauma | "...exposure to actual or threatened death, serious injury, or sexual violence, and can be experienced directly or by witnessing such an event…" | Self-completed questionnaire using the Childhood Trauma Questionnaire. | NR | 5 | emotional abuse; physical abuse; sexual abuse; emotional neglect; physical neglect | Cases: Total score: median 57.5; mean 62.6; SD 27.0* Emotional abuse score: median 11.5; mean 13.0; SD 6.7* Physical abuse score: median 11.0; mean 12.6; SD 6.7* Sexual abuse score: median 7.5; mean 10.0; SD 6.1* Emotional neglect score: median 12.5; mean 14.2; SD 6.9* Physical neglect score: median 9.0; mean 11.1; SD 5.8*  Controls: Total score: median 31.0; mean 34.1; SD 9.0* Emotional abuse score: median 5.0; mean 7.4; SD 3.6* Physical abuse score: median 5.0; mean 5.8; SD 1.6* Sexual abuse score: median 5.0; mean 5.6; SD 2.5* Emotional neglect score: median 7.0; mean 7.8; SD 3.2* Physical neglect score: median 5.0; mean 6.6; SD 2.6* |
| Garland 2019 | Adverse childhood experiences | NR | Self-completed questionnaire using the Adverse Childhood Experiences Questionnaire. | NR | 10 | sexual abuse; emotional abuse; physical abuse; emotional neglect; physical neglect; parental separation/divorce; domestic violence; household substance misuse; household mental illness; household member incarceration | Mean 2.8; SD 2.3  0: 6/36 (16.7%) ≥1: 30/36 (83.3%)  Sexual abuse: 15/36 (41.7%) Emotional abuse: 13/36 (36.1%) Physical abuse: 9/36 (25.0%) Emotional neglect: 13/36 (36.1%) Physical neglect: 6/36 (16.7%) Parental separation or divorce: 10/36 (27.8%) Battered mother: 6/36 (16.7%) Substance abuse in household member: 9/36 (25.0%) Mental illness in household member: 16/36 (44.4%) Incarcerated household member: 2/36 (5.6%) |
| Griego 2022 | Adverse childhood experiences | "...potentially traumatic events that occur in childhood, including experiences of violence, abuse, or exposure to household substance use disorders…" | Self-completed questionnaire using the Behavioral Risk Factor Surveillance System ACE module. | NR | NR | List not reported. Source of the list of ACEs was cited as Merrick et al. 2018, who cited Ford et al. 2014, who measured 9 ACEs using 11 questions: household mental illness; household alcohol misuse; household substance misuse; household member incarceration; parental separation/divorce; domestic violence; physical abuse; emotional abuse; sexual abuse | NR |
| Groh 2020 | Childhood trauma | NR | Self-completed questionnaire using the Childhood Trauma Questionnaire. | NR | 5 | emotional abuse; physical abuse; sexual abuse; emotional neglect; physical neglect | Emotional abuse score: mean 11.5* Physical abuse score: mean 10.7* Sexual abuse score: mean 7.7* Emotional neglect score: mean 15.1* Physical neglect score: mean 9.9* |
| Guarino 2021 | Adverse childhood experiences | NR | Structured interview using the Adverse Childhood Experiences Questionnaire. | < 18 | 10 | household mental illness; household member incarceration; parental separation/divorce; emotional neglect; household alcohol/substance misuse; physical neglect; domestic violence; sexual abuse; physical abuse; emotional abuse | 0: 58/539 (10.8%) 1-3: 234/539 (43.4%) 4-6: 159/539 (29.5%) 7-10: 88/539 (16.3%)  Household mental illness: 330/539 (61.2%) Household member incarceration: 283/539 (52.5%) Parental separation/divorce: 243/539 (45.1%) Emotional neglect: 229/539 (42.5%) Household substance misuse: 188/539 (34.9%) Physical neglect: 183/539 (34.0%) Domestic violence: 139/539 (25.8%) Sexual abuse: 128/539 (23.7%) Physical abuse: 106/539 (19.7%) Emotional abuse: 89/539 (16.5%) |
| Heffernan 2000 | Childhood abuse | Childhood sexual abuse: "...non-consensual sexual bodily contact ranging from fondling to penetration prior to age 16 by someone of any age or relationship to the patient…"  Childhood physical abuse: "...being hit, punched, cut, or pushed down by an adult in charge of the patient (e.g., parent, stepparent, foster par- ent) in such a way that this resulted in injury..." | Structured interview using author determined questions. | < 16 | 2 | sexual abuse; physical abuse | 0: 446*/763 (58.5%) 1: 204*/763 (26.7%) 2: 113*/763 (14.8%)  Physical abuse: 244*/763 (32.0%*) Sexual abuse: 184*/763 (24.1%*) |
| Hill 2022 | Childhood maltreatment | "...the experience of emotional or physical neglect and/or abuse within the family…" | Self-completed questionnaire using the Childhood Trauma Questionnaire. | NR | 5 | emotional abuse; physical abuse; sexual abuse; emotional neglect; physical neglect | Mean childhood trauma questionnaire score 32.6; SD 8.2 |
| Khoury 2010 | Childhood traumatic experiences | "...such as physical and sexual abuse as well as neglect…" | Structured interview using the Early Trauma Inventory. | NR | 3 | physical abuse; sexual abuse; emotional abuse | Physical abuse score: mean 35.4; SD 49.5 Sexual abuse score: mean 33.2; SD 85.0 Emotional abuse score: mean 55.2, SD 74.5 |
| Kors 2022 | Childhood maltreatment | NR | Self-completed questionnaire using the Maltreatment and Abuse Chronology of Exposure questionnaire. | NR | 4 | sexual abuse; physical abuse; emotional abuse; neglect | NR |
| Kumar 2016 | Childhood trauma | NR | Initial clinical assessment using the Childhood Trauma Questionnaire. | NR | 5 | physical abuse; sexual abuse; emotional abuse; physical neglect; emotional neglect | Less trauma: 95*/113 (84.1%) Moderate/severe trauma: 18/113 (15.9%)  Moderate/severe physical abuse: 22/113 (19.5%) Moderate/severe sexual abuse: 18/113 (15.9%) Moderate/severe emotional abuse: 28/113 (24.8%) Moderate/severe physical neglect: 26/113 (23.0%) Moderate/severe emotional neglect: 20/113 (17.7%) |
| Larance 2018 | Childhood maltreatment | NR | Structured interview using questions from the Christchurch Health and Development Study, supplemented with additional author determined questions. | < 18 | 4 | physical abuse; sexual abuse; emotional abuse; neglect | 0: 273/1149 (23.8%) 1: 270/1149 (23.5%) 2: 247/1149 (21.5%) 3: 222/1149 (19.3%) 4: 137/1149 (11.9%)  Emotional abuse: 597/1149 (52.0%) Physical abuse: 563/1149 (49.0%) Sexual abuse: 552/1149 (48.0%) Neglect: 471/1149 (41.0%) |
| Lee 2023 | Childhood abuse | NR | Self-completed questionnaire using questions from the Childhood Trauma Questionnaire. | NR | 3 | emotional abuse; physical abuse; sexual abuse | Emotional abuse score: mean: 0.68*; SD 0.60* Physical abuse score: mean 0.60*; SD 0.54* Sexual abuse score: mean 0.29*; SD 0.57* |
| Lovallo 2018 | Early life adversity | "...usually assessed by a reported history of physical and sexual abuse and neglect, and also by low socioeconomic status…" | Clinical interview with items from the post-traumatic stress disorders module of the Computerized Diagnostic Interview Schedule Version IV (C-DIS-IV). | < 15 | 4 | physical adversity; sexual adversity; emotional adversity; socioeconomic status | Used early life adversity score rather than ACE count |
| Lynskey 2006 | Childhood sexual abuse; childhood physical abuse | NR | Structured telephone interview using author determined questions. | < 18 | 2 | sexual abuse; physical abuse | Sexual abuse: females 17.0%, males 5.7% Physical abuse: females 4.2%, males 2.9% |
| Martin 2023 | Adverse childhood experiences | "...a broad array of experiences including parental divorce; problematic alcohol or drug use, mental illness, or suicide attempts in the home; family member incarceration; physical, sexual, or emotional abuse; and lack of emotional support or adequate food, clothing, or medical care..." | Self-completed questionnaire using the Adverse Childhood Experiences Questionnaire. | NR | 4 | emotional/physical abuse; household dysfunction; sexual abuse; neglect | 0: 141/565 (25.0%) 1-3: 268/565 (47.4%) ≥4: 156/565 (27.6%)  Emotional/physical abuse: 212/565 (37.6%) Household dysfunction: 383/565 (67.8%) Sexual abuse: 109/565 (19.4%) Neglect: 135/565 (24.0%) |
| McDonagh 2023 | Adverse childhood experiences | NR | Structured interview using the Adverse Childhood Experiences Questionnaire. | NR | 10 | emotional abuse; physical abuse; sexual abuse; emotional neglect; physical neglect; parental separation/divorce; domestic violence; household alcohol/substance misuse; household mental illness/suicide; household member incarceration | Mean 4; SD 2.9  0: 12/104 (11.5%) 1-3: 34/104 (32.7%) ≥4: 57/104 (54.8%)  Emotional abuse: 45/104 (43.3%) Physical abuse: 41/104 (39.4%) Sexual abuse: 37/104 (35.6%) Emotional neglect: 37/104 (35.6%) Physical neglect: 27/104 (26.0%) Parental divorce/separation: 42/104 (40.4%) Domestic violence: 37/104 (35.6%) Household alcohol/substance misuse: 61/104 (58.7%) Household mental illness/suicide: 46/104 (44.2%) Household member incarceration: 47/104 (45.2%) |
| Meadows 2023 | Adverse childhood experiences | NR | Self-completed questionnaire using the Adverse Childhood Experiences Questionnaire. | < 18 | 10 | emotional abuse; physical abuse; sexual abuse; physical neglect; emotional neglect; domestic violence; household substance misuse; parental separation/divorce; household mental illness; household member incarceration | Mean 2.3; SD 2.2  ≥4: 26*/107 (24.3%) |
| Merrick 2020 | Adverse childhood experiences | "...child abuse and neglect and various forms of household challenges… | Montana: Self-completed questionnaire using the Behavioral Risk Factor Surveillance System ACE module.  Florida: NR. | < 18 | Montana: 8  Florida: 5 | Montana: emotional abuse; physical abuse; sexual abuse; household mental illness; household substance misuse; domestic violence; parental separation/divorce; household member incarceration  Florida: parental divorce or death; household substance misuse; household mental illness; domestic violence; any child abuse | Montana: 0: 3255*/8726 (37.3%) 1-2: 3168*/8726 (36.3%) ≥3: 2303*/8726 (26.4%)  Florida: 0: 13773*/27545 (50.0%) 1-2: 10329*/27545 (37.5%) ≥3: 3388*/27545 (12.3%) |
| Mirhashem 2017 | Childhood maltreatment | "...the range of abuse and neglect children experience from caregivers, including emotional abuse, emotional neglect, physical abuse, physical neglect, and sexual abuse…" | Self-completed questionnaire using the Childhood Trauma Questionnaire. | NR | 5 | emotional neglect; emotional abuse; physical neglect; physical abuse; sexual abuse | NR |
| Myers 2014 | Childhood adversity | NR | Structured interview using questions from Conflict Tactics Scale and the Childhood Trauma Questionnaire. | < 18 | 9 | emotional abuse; physical abuse; domestic violence; neglect; endangerment; sexual abuse; parental mental illness; parental incarceration; parental alcohol/substance misuse | 0: 18919/34653 (56.3% weighted) 1-2: 10016/34653 (28.3% weighted) ≥3: 5718/34653 (15.4% weighted) |
| Naqavi 2011 | Childhood maltreatment | NR | Self-completed paper questionnaire using the Childhood Trauma Questionnaire. | NR | 5 | sexual abuse; physical abuse; emotional abuse; physical neglect; emotional neglect | NR |
| Nelson 2006 | Childhood sexual abuse | NR | Structured telephone interview using author determined questions. | < 18 | 1 | sexual abuse | Sexual abuse: females 17.4%, males 6.0% |
| Onu 2021 | Adverse childhood experiences | "…the intensive and frequently occurring sources of stress experienced by children early in life, such as multiple types of abuse, neglect, violence between parents or caregivers, and other kinds of serious household dysfunction such as alcohol and substance abuse, and peer, community, and collective violence..." | Self-completed questionnaire using the Adverse Childhood Experiences Questionnaire. | < 18 | 10 | physical abuse; emotional abuse; sexual abuse; physical neglect; emotional neglect; household substance misuse; parental separation/divorce; domestic violence  Full list not reported. Source of the list of ACEs was cited as Felitti et al. 1998, who measured 7 ACEs using 17 questions, and did not measure physical neglect, emotional neglect or parental separation/divorce, but did measure household mental illness and household member incarceration. | Mean 3.8; SD 2.2 |
| Pakdaman 2021 | Adverse childhood experiences | "...traumatic events that occur prior to age 18…" | Self-completed online questionnaire using 9 items derived from the Adverse Childhood Experiences Questionnaire. | < 18 | 9 | parental substance misuse; parental mental illness; physical abuse; sexual abuse; emotional abuse; homelessness  Full list not reported. Source of the list of ACEs was cited as Felitti et al. 1998, who measured 7 ACEs using 17 questions, and did not measure homelessness. | 0: NR 1: 686/3899 (17.6%) 2: 408/3899 (10.5%) 3: NR ≥4: 349/3899 (9.0%) |
| Pierce 2019 | History of abuse | "Patients were asked: Do you have a history of physical or sexual abuse…were asked to indicate when this occurred…" | Self-completed paper questionnaire using author determined questions. | < 19 | 1 | physical or sexual abuse | Childhood physical or sexual abuse: 205/1785 (11.5%) |
| Pierce 2020 | Child abuse | "…do you have a history of physical or sexual abuse?..." | Self-completed paper questionnaire using author determined questions. | < 19 | 1 | child abuse | 0: 2725*/3118 (87.4%) 1: 393*/3118 (12.6%) |
| Quinn 2016 | Childhood trauma | NR | Structured interview using Add Health cohort questions. | < 18 | 9 | neglect; emotional abuse; physical abuse; sexual abuse; parental incarceration; witnessed violence; threatened with violence; experienced violence; parental binge drinking | 0: 4477/9569 (47.2% weighted) 1: 2677/9569 (28.1% weighted) 2: 1307/9569 (13.3% weighted) 3: 646/9569 (6.7% weighted) 4: 297/9569 (3.0% weighted) ≥5: 165/9569 (1.8% weighted)  Neglect: 1550/12288 (12.7% weighted) Emotional abuse: 1981/12288 (16.4% weighted) Physical abuse: 1477/12288 (12.2% weighted) Sexual abuse: 984/12288 (8.3% weighted) Parental incarceration: 1279/12288 (10.4% weighted) Parental binge drinking: 1271/12288 (13.1% weighted) Witnessed violence: 1445/12288 (11.0% weighted) Threatened with violence: 1490/12288 (12.1% weighted) Experienced violence: 619/12288 (5.1% weighted) |
| Roy 2002 | Childhood trauma | NR | Self-completed questionnaire using the Childhood Trauma Questionnaire | NR | 5 | emotional abuse; physical abuse; sexual abuse; emotional neglect; physical neglect | Emotional abuse score: mean 11.0*; SD 5.8* Physical abuse score: mean 10.7*; SD 3.6* Sexual abuse score: mean 9.4*; SD 5.4* Emotional neglect score: mean 24.5*; SD 10.2* Physical neglect score: mean 14.9*; SD 5.9* |
| Sansone 2010 | Childhood trauma | NR | Self-completed paper questionnaire using author determined questions. | < 12 | 5 | sexual abuse; physical abuse; emotional abuse; physical neglect; witnessed violence | Mean 1.6; SD 1.7 |
| Santo Jr 2022 | Childhood trauma | "...the experience of a potentially distressing event(s) that threatens a child’s health or integrity…" | Structured interview using "…five binary questions…adapted from Sansone et al. (2009)…", with subsequent latent class analysis identifying 3 classes. | < 17 | 5 | sexual abuse; physical abuse; emotional abuse; physical neglect; witnessed violence | 0: 773*/1514 (51.1%) ≥1: 741*/1514 (48.9%)  Emotional abuse: 560*/1514 (37.0%) Witnessed violence: 530*/1514 (35.0%) Physical abuse: 439*/1514 (29.0%) Sexual abuse: 333*/1514 (22.0%) Physical neglect: 197*/1514 (13.0%) |
| Sartor 2014 | Childhood risk factors | NR | Structured interview using author determined questions. | < 14 | 7 | death of a parent age <6; witnessed violent crime; victim of violent crime; sexual abuse; severe physical abuse; household cocaine misuse; household heroin misuse | Death of a parent age <6: 97*/2158* (4.5%*) Witnessed violent crime: 481*/2158 (22.3%*) Victim of violent crime: 178*/2158* (8.3%*) Sexual abuse: 399*/2158* (18.5%*) Severe physical abuse: 262*/2158* (12.1%*) Household cocaine use: 247*/2158* (11.4%*) Household heroin use: 147*/2158* (6.8%*) |
| Smith 2022 | Adverse childhood experiences | NR | Self-completed online questionnaire using the Adverse Childhood Experiences Questionnaire. | < 18 | 10 | List not reported. Source of the list of ACEs was cited as Dong et al. 2004, who measured 10 ACEs using 28 questions.  emotional abuse; physical abuse; sexual abuse; emotional neglect; physical neglect; domestic violence; household alcohol/substance misuse; household mental illness/suicide; parental separation/divorce; household member incarceration | Mean 2.7; SD 2.6 |
| Stein 2017 | Adverse childhood experiences | NR | Structured interview using the Adverse Childhood Experiences Questionnaire. | < 18 | 10 | emotional abuse; physical abuse; sexual abuse; emotional neglect; physical neglect; parental separation/divorce; domestic violence; household alcohol/substance misuse; household mental illness/suicide; household member incarceration | Mean 3.64 (SD 2.75)  0: 67/457 (14.7%) 1-3: 168/457 (36.8%) ≥4: 222/457 (48.6%)  Emotional abuse: 218*/457 (47.7%) Physical abuse: 165*/457 (36.2%) Sexual abuse: 90*/457 (19.6%) Emotional neglect: 177*/457 (38.8%) Physical neglect: 75*/457 (16.5%) Parental separation/divorce: 273*/457 (59.8%) Domestic violence: 132*/457 (28.8%) Household alcohol/substance misuse: 234*/457 (51.3%) Hosuehold mental illness/suicide: 157*/457 (34.4%) Household member incarceration: 93*/457 (20.3%) |
| Tang 2020 | Adverse childhood experiences | "...a collection of potentially, traumatic experiences that occur during the first 18 years of life…" | Structured interview using questions from Conflict Tactics Scale and the Childhood Trauma Questionnaire. | < 18 | 10 | emotional abuse; physical abuse; sexual abuse; physical neglect; emotional neglect; domestic violence; household substance misuse; household member incarceration; household mental illness; parental separation/divorce | Mean 1.3; SD 1.8 |
| Taplin 2014 | Childhood trauma | NR | Structured interview using the Childhood Trauma Questionnaire | < 18 | 5 | emotional abuse; physical abuse; sexual abuse; emotional neglect; physical neglect | 0: 27.1% ≥1 moderate/extreme ACE: 62/87 (71.3%)  Emotional abuse: 40/87 (46.0%) Physical abuse: 36/87 (41.4%) Sexual abuse: 35/87 (40.2%) Physical neglect: 31/87 (35.6%) Emotional neglect: 40/87 (46.0%) |
| Testa 2023 | Adverse childhood experiences | "...experiences with abuse, neglect, and household dysfunction during childhood and adolescence…" | Self-completed paper questionnaire or structured interview using the Adverse Childhood Experiences Questionnaire. | < 18 | 10 | parental separation/divorce; household alcohol/substance misuse; household mental illness; household member incarceration; sexual abuse; emotional abuse; physical abuse; emotional neglect; physical neglect; domestic violence | 0: 1184*/2999 (39.5%) 1: 570*/2999 (19.0%) 2: 345*/2999 (11.5%) ≥3: 900*/2999 (30.0%) |
| Thiesset 2023 | Adverse childhood experiences | NR | Self-completed online questionnaire using the Adverse Childhood Experiences Questionnaire. | NR | 10 | physical neglect; loss of parent; household mental illness; household alcohol/substance misuse; domestic violence; household member incarceration; emotional abuse; physical abuse; emotional neglect; sexual abuse | <4: 62/117 (53.0%) ≥4: 55/117 (47.0%)  Physical neglect: 27/116 (23.3%) Loss of parent: 53/117 (45.3%) Household member mental illness: 64/117 (54.7%) Household member alcohol/substance misuse: 50/117 (42.7%) Witnessed domestic violence: 28/117 (23.9%) Household member incarceration: 26/117 (22.2%) Emotional abuse: 46/99 (46.5%) Physical abuse: 50/117 (42.7%) Emotional neglect: 38/116 (32.8%) Sexual abuse: 35/116 (30.2%) |
| Tomassi 2017 | Childhood trauma | NR | Structured interview using the Childhood Experience of Care and Abuse Questionnaire. | < 16 | 3 | severe sexual abuse; severe physical abuse; separation from or death of parent | 0: 217*/345 (62.9%*) ≥1: 128*/345 (36.8%*)  Severe sexual abuse: 27*/345 (7.8%*) Severe physical abuse: 48*/345 (13.9%*) Separation from or death of parent: 69*/345 (20.0%*) |
| Vogel 2011 | Traumatic childhood experiences | NR | Self-completed questionnaire using the Childhood Trauma Questionnaire. | NR | 5 | emotional abuse; physical abuse; sexual abuse; emotional neglect; physical neglect | Childhood trauma questionnaire score: Median 45; IQR 36-62 |
| Wang 2021 | Adverse childhood experiences | "...any early negative childhood experiences (e.g. physical and sexual abuse, parental divorce, and emotional and physical neglect)…" | Structured interview using the Adverse Childhood Experiences Questionnaire. | NR | 10 | emotional abuse; physical abuse; sexual abuse; domestic violence; household mental illness; parental separation/divorce; household member incarceration; household substance misuse; emotional neglect; physical neglect | Mean 1.46; SD 1.90  0: 13632/33613 (42.2%) 1: 8090/33613 (23.9%) 2: 4320/33613 (12.4%) ≥3: 7571/33613 (21.5%) |
| Widom 2006 | Child abuse and/or neglect | "...court-substantiated cases of child abuse and neglect…" | Records of county juvenile and adult criminal courts. | < 12 | 1 | abuse/neglect | Abuse/neglect: 496*/892 (55.6%*) |
| Williams 2020 | Adverse childhood experiences | NR | Self-completed online questionnaire using the Adverse Childhood Experiences Questionnaire. | NR | 17 | emotional abuse; physical abuse; sexual abuse; domestic violence; household substance misuse; household mental illness/suicide; household member incarceration  Full list not reported. Source of the list of ACEs was cited as Felitti et al. 1998, who measured 7 ACEs using 17 questions. | Mean 6.3; SD 4.5 |
| Williams 2021 | Adverse childhood experiences | NR | Self-completed online questionnaire using the Adverse Childhood Experiences Questionnaire. | NR | 17 | emotional abuse; physical abuse; sexual abuse; domestic violence; household substance misuse; household mental illness/suicide; household member incarceration  Full list not reported. Source of the list of ACEs was cited as Felitti et al. 1998, who measured 7 ACEs using 17 questions. | Mean 6.4; SD 4.5 |
| Wuest 2007 | Abused as a child | NR | Structured interview with a single question: "were you abused as a child?". | NR | 1 | abuse | Abuse: 205*/309 (66.3%) |
| You 2019 | Childhood adversity | NR | Self-completed online questionnaire using the Early Traumatic Inventory Self-Report. | < 18 | 27 | general trauma; physical trauma; emotional trauma; sexual trauma  Asked 27 questions on 4 trauma themes. | Interquartile range 3-8; median 5; mean 5.7; SD 4.1  0: 246*/3073 (8.0%) ≥1: 2827*/3073 (92.0%)  General trauma: 2397*/3073 (78.0%) Physical trauma: 2243*/3073 (73.0%) Emotional trauma: 1352*/3073 (44.0%) Sexual trauma: 615*/3073 (20.0%) |
| Zehetmeier 2023 | Adverse childhood experiences | NR | Self-completed questionnaire using the Childhood Trauma Questionnaire. | NR | 5 | emotional abuse; physical abuse; sexual abuse; emotional neglect; physical neglect | Emotional abuse: 50/191 (26.2%*) Physical abuse: 24/191 (12.6%*) Sexual abuse: 25/191 (13.1%*) Emotional neglect: 52/191 (27.2%*) Physical neglect: 27/191 (14.1%*) |

ACE = adverse childhood experience, NR = not reported, SD = standard deviation. *Value calculated from data presented in paper.

Table S6: Commonly assessed adverse childhood experiences

| Adverse Childhood Experience | Total Studies | Studies | Studies Included in Meta-Analysis  of Prevalence | Pooled Prevalence | Heterogeneity |
| --- | --- | --- | --- | --- | --- |
| Sexual abuse | 55 | Afifi 2012; Alexander 1998; Austin 2018a; Austin 2018b; Baumann-Larsen 2023; Bottiroli 2019; Browne 1998; Carlyle 2021; Carlyle 2023; Carr 2023; Conroy 2009; Davis 2022; Derefinko 2019; Eaves 2021; El-Bassel 2019; Elhammady 2014; Fortson 2021; Garami 2019; Garland 2019; Groh 2020; Guarino 2021; Heffernan 2000; Hill 2022; Khoury 2010; Kors 2022; Kumar 2016; Larance 2018; Lee 2023; Lynskey 2006; Martin 2023; McDonagh 2023; Meadows 2023; Merrick 2020; Mirhashem 2017; Myers 2014; Naqavi 2011; Nelson 2006; Onu 2021; Pakdaman 2021; Quinn 2016; Roy 2002; Sansone 2010; Santo Jr 2022; Sartor 2014; Stein 2017; Tang 2020; Taplin 2014; Testa 2023; Thiesset 2023; Tomassi 2017; Vogel 2011; Wang 2021; Williams 2020; Williams 2021; Zehetmeier 2023 | 20 | 20.6% (95% CI 15.1-27.6%) | I^2^ = 99.2%, Cochran Q = 2,409, p < 0.001 |
| Physical abuse | 52 | Afifi 2012; Alexander 1998; Austin 2018a; Austin 2018b; Bottiroli 2019; Browne 1998; Carlyle 2021; Carlyle 2023; Carr 2023; Conroy 2009; Davis 2022; Derefinko 2019; Eaves 2021; El-Bassel 2019; Elhammady 2014; Fortson 2021; Garami 2019; Garland 2019; Groh 2020; Guarino 2021; Heffernan 2000; Hill 2022; Khoury 2010; Kors 2022; Kumar 2016; Larance 2018; Lee 2023; Lynskey 2006; McDonagh 2023; Meadows 2023; Merrick 2020; Mirhashem 2017; Myers 2014; Naqavi 2011; Onu 2021; Pakdaman 2021; Quinn 2016; Roy 2002; Sansone 2010; Santo Jr 2022; Sartor 2014; Stein 2017; Tang 2020; Taplin 2014; Testa 2023; Thiesset 2023; Tomassi 2017; Vogel 2011; Wang 2021; Williams 2020; Williams 2021; Zehetmeier 2023 | 16 | 28.6% (95% CI 22.8-35.1%) | I^2^ = 98.3%, Cochran Q = 886, p < 0.001 |
| Emotional abuse | 44 | Afifi 2012; Austin 2018a; Bottiroli 2019; Carlyle 2021; Carlyle 2023; Carr 2023; Conroy 2009; Davis 2022; Derefinko 2019; Eaves 2021; El-Bassel 2019; Fortson 2021; Garami 2019; Garland 2019; Groh 2020; Guarino 2021; Hill 2022; Khoury 2010; Kors 2022; Kumar 2016; Larance 2018; Lee 2023; McDonagh 2023; Meadows 2023; Merrick 2020; Mirhashem 2017; Myers 2014; Naqavi 2011; Onu 2021; Pakdaman 2021; Quinn 2016; Roy 2002; Sansone 2010; Santo Jr 2022; Stein 2017; Tang 2020; Taplin 2014; Testa 2023; Thiesset 2023; Vogel 2011; Wang 2021; Williams 2020; Williams 2021; Zehetmeier 2023 | 13 | 38.2% (95% CI 30.1-46.2%) | I^2^ = 97.8%, Cochran Q = 554, p < 0.001 |
| Physical neglect | 29 | Afifi 2012; Austin 2018b; Bottiroli 2019; Carlyle 2021; Carlyle 2023; Carr 2023; Fortson 2021; Garami 2019; Garland 2019; Groh 2020; Guarino 2021; Hill 2022; Kumar 2016; McDonagh 2023; Meadows 2023; Mirhashem 2017; Naqavi 2011; Onu 2021; Roy 2002; Sansone 2010; Santo Jr 2022; Stein 2017; Tang 2020; Taplin 2014; Testa 2023; Thiesset 2023; Vogel 2011; Wang 2021; Zehetmeier 2023 | 10 | 17.7% (95% CI 12.2-25.0%) | I^2^ = 96.8%, Cochran Q = 279, p < 0.001 |
| Emotional neglect | 25 | Afifi 2012; Bottiroli 2019; Carlyle 2021; Carlyle 2023; Fortson 2021; Garami 2019; Garland 2019; Groh 2020; Guarino 2021; Hill 2022; Kumar 2016; McDonagh 2023; Meadows 2023; Mirhashem 2017; Naqavi 2011; Onu 2021; Roy 2002; Stein 2017; Tang 2020; Taplin 2014; Testa 2023; Thiesset 2023; Vogel 2011; Wang 2021; Zehetmeier 2023 | 8 | 33.5% (95% CI 27.3-40.3%) | I^2^ = 95.2%, Cochran Q = 146, p < 0.001 |
| Domestic violence | 19 | Carr 2023; Davis 2022; Eaves 2021; El-Bassel 2019; Fortson 2021; Garland 2019; Guarino 2021; McDonagh 2023; Meadows 2023; Merrick 2020; Myers 2014; Onu 2021; Stein 2017; Tang 2020; Testa 2023; Thiesset 2023; Wang 2021; Williams 2020; Williams 2021 | 8 | 25.2% (95% CI 16.5-36.5%) | I^2^ = 97.1%, Cochran Q = 245, p < 0.001 |
| Household member incarceration | 17 | Carr 2023; Eaves 2021; Fortson 2021; Garland 2019; Guarino 2021; McDonagh 2023; Meadows 2023; Merrick 2020; Myers 2014; Quinn 2016; Stein 2017; Tang 2020; Testa 2023; Thiesset 2023; Wang 2021; Williams 2020; Williams 2021 | 8 | 23.3% (95% CI 13.0-38.3%) | I^2^ = 98.5%, Cochran Q = 456, p < 0.001 |
| Household mental illness | 17 | Carr 2023; Eaves 2021; Fortson 2021; Garland 2019; Guarino 2021; McDonagh 2023; Meadows 2023; Merrick 2020; Myers 2014; Pakdaman 2021; Stein 2017; Tang 2020; Testa 2023; Thiesset 2023; Wang 2021; Williams 2020; Williams 2021 | 8 | 40.1% (95% CI 32.1-48.6%) | I^2^ = 95.6%, Cochran Q = 159, p < 0.001 |
| Parental separation/divorce | 13 | Carr 2023; Eaves 2021; Fortson 2021; Garland 2019; Guarino 2021; McDonagh 2023; Meadows 2023; Merrick 2020; Onu 2021; Stein 2017; Tang 2020; Testa 2023; Wang 2021 | 7 | 46.0% (95% CI 36.8-55.6%) | I^2^ = 96.4%, Cochran Q = 167, p < 0.001 |
| Household substance misuse | 11 | Carr 2023; Eaves 2021; Garland 2019; Meadows 2023; Merrick 2020; Onu 2021; Pakdaman 2021; Tang 2020; Wang 2021; Williams 2020; Williams 2021 | 2 | 43.5% (95% CI 33.5-54.1%) | I^2^ = 81.6%, Cochran Q = 5, p = 0.020 |
| Household alcohol/ substance misuse | 8 | Elhammady 2014; Fortson 2021; Guarino 2021; McDonagh 2023; Myers 2014; Stein 2017; Testa 2023; Thiesset 2023 | 6 | 37.7% (95% CI 27.2-49.5%) | I^2^ = 97.4%, Cochran Q = 195, p < 0.001 |
| Neglect | 7 | Conroy 2009; Derefinko 2019; Kors 2022; Larance 2018; Martin 2023; Myers 2014; Quinn 2016 | 2 | 32.0% (95% CI 21.3-44.9%) | I^2^ = 97.9%, Cochran Q = 47, p < 0.001 |
| Witnessed violence | 5 | Baumann-Larsen 2023; Quinn 2016; Sansone 2010; Santo Jr 2022; Sartor 2014 | 2 | 26.6% (95% CI 17.3-38.5%) | I^2^ = 99.2%, Cochran Q = 124, p < 0.001 |

Table 1: The most commonly assessed ACEs (only ACEs included in ≥3 studies are presented). Where sufficient data were reported, studies were combined to give an estimate of the pooled prevalence of each ACE. ACE = adverse childhood experience, CI = confidence interval.
